# LncRNA MEG3 promotes cisplatin sensitivity of cervical cancer cells by regulating the miR-21/PTEN axis

**DOI:** 10.1186/s12885-022-10188-0

**Published:** 2022-11-07

**Authors:** Ying Du, Gang Geng, Chunquan Zhao, Tian Gao, Bin Wei

**Affiliations:** 1 Department of Gynecology, The First Affiliated Hospital of Chongqing Medical University, Chongqing, 400016 China; 2 Department of Respiratory, Children’s Hospital of Chongqing Medical University, Chongqing, 400014 China; 3 Department of Dermatology, The First Affiliated Hospital of Chongqing Medical University, No. 1 Youyi Road, Yuzhong District, Chongqing, 400016 China

**Keywords:** Cervical cancer, MEG3, miR-21, PTEN, Cisplatin; chemosensitivity

## Abstract

**Background:**

Cervical cancer (CC) is a common gynecological malignancy worldwide. Some patients perform serious resistance after chemotherapy, and long-stranded non-coding RNA MEG3 is reported to be involved in the regulation of chemoresistance in many solid tumors. However, its involvement in cervical adenocarcinoma has not been reported.

**Methods:**

Hela cell lines, cisplatin-resistant cell lines (Hela-CR) and nude mice were used in this study. After MEG3 was overexpressed or knocked down in cells by the lentivirus vector, cell growth was detected by the CCK-8 assay, and cell migration was evaluated using Transwell assay. Quantitative real-time polymerase chain reaction (qRT-PCR) was performed to examine the expression of MEG3, miR-21 and PTEN mRNA. Apoptosis was detected by flow cytometry. The targeting relationship between mRNAs was predicted and verified using dual-luciferase reporter gene experiments. Western blot was executed to examine Bax, cleaved-caspase 3, Bcl-2, PTEN and GAPDH expression. Cells were injected into the mice to form xenograft tumors to compare tumorigenesis capacity.

**Results:**

We demonstrated that MEG3 was down-regulated in cervical cancer by analyzing the TCGA database. Moreover, knockdown of MEG3 promoted CC cell proliferation, migration and inhibited the apoptosis. These changes of CC cells were more pronounced under cisplatin treatment. Further studies showed that the MEG3/miR-21/PTEN axis affected cisplatin sensitivity in cervical cancer cells, and these results of recue assay were used to confirm this conclusion.

**Conclusions:**

MEG3 performing as ceRNA promotes cisplatin sensitivity in CC cells through the miR-21/PTEN axis.

**Supplementary Information:**

The online version contains supplementary material available at 10.1186/s12885-022-10188-0.

## Background

Cervical cancer (CC) is one of the common gynaecological cancers and its incidence rate is the third highest among female cancers worldwide [1]. In China, the incidence of cervical cancer is about 96,000 new cases in 2015, accounting for about 18% of new cases of cervical cancer in the world, including about 26,000 deaths [2]. CC has become a great threat to women’s health. Cervical adenocarcinoma accounts for about 20–25% of the different pathological types, and its incidence is increasing year by year, and the incidence age of CC patients gradually gets younger [3]. Chemotherapy is one of the most important treatments for patients with large localized lesions or systemic metastases [4]. Chemotherapy can improve patients’ prognosis, reduce localized lesions, facilitate subsequent treatment, and inhibit distant metastases [5]. And paclitaxel in combination with cisplatin is the first-line chemotherapy regimen for cervical adenocarcinoma, however, some patients have poor outcome due to chemoresistance, mainly cisplatin resistance, leading to dismal prognosis [6]. Therefore, it is important to investigate the mechanism of cisplatin resistance in cervical adenocarcinoma and improve cisplatin sensitivity in the treatment of cervical adenocarcinoma.

Numerous studies have confirmed that long non-coding RNA (lncRNA) is involved in chemoresistance in a variety of malignancies and plays a key regulatory role [7–10]. The human maternally expressed gene 3 (MEG3) is localized in the MEG3 imprinted region of 14q32.3 delta-like 1 homologue (DLK1) and contains multiple imprinted genes that are widely distributed in brain, adrenal, placenta, ovary, spleen, breast. MEG3 is significantly downregulated in primary human cancers such as lung cancer, liver cancer, gallbladder cancer, pituitary tumors and different cancer cell lines, and its expression level is significantly related with tumor grade, metastasis and poor prognosis [11]. Furthermore, the molecular mechanism investigation revealed that reduced expression of MEG3 resulted in increased drug resistance in a variety of malignancies [12, 13]. Exogenous overexpression of MEG3 was reported to lead to tumor cells to regain drug sensitivity, suggesting that MEG3 may regulate the biological activity of tumor cells by modulating their drug sensitivity [14]. However, whether Lnc RNA MEG3 is involved in chemoresistance in cervical adenocarcinoma has not been reported.

In this study, we investigated the effect and mechanism of MEG3 on cisplatin sensitivity in CC cells to provide a theoretical basis for using MEG3 as a target for CC therapy.

## Materials and methods

### Cell culture

The CC cell line Hela was purchased from the Cell Bank of the Chinese Academy of Sciences (Shanghai, China). HeLa cell were cultured in increasing concentrations of cisplatin for over 6 months to establish cisplatin‐resistant (CR) cell lines (Hela-CR). Cells were cultured in DMEM with 10% fetal bovine serum, 100 U/ml penicillin and 100 U/ml streptomycin at 37 °C in a 5% CO_2_ incubator.

### Cell Transfection

The lentivirus vector containing MEG3 knockdown or MEG3 overexpression lentivirus or the corresponding negative control were purchased from Shanghai Gene Pro Technology Co. Lentiviral transfection was performed strictly according to the instructions. Briefly, 3 × 10^3^ cells were inoculated in 6-well culture plates and after the degree of cell fusion reached 50%, the virus and 5 µg/ml Polybrene were added to the complete medium, the plates were put back into the cell incubator and the culture was continued for 24 h and then replaced with fresh medium and the culture was continued. Cell lines with stable transfection were obtained.

mimic NC (B04001, GenePharma, China), miR-21 mimic (B01001, GenePharma, China), inhibitor NC (B04003, GenePharma, China), miR-21 inhibitor (B03001, GenePharma, China), siNC (A06001, GenePharma, China), siPTEN (A01004, GenePharma, China), pcDNA3.1 empty vector and pcDNA3.1-PTEN overexpression plasmid were purchased from Shanghai GenePharma Technology Co. The targeted sequence of PTEN siRNA is 5’-AACAGTAGAGGAGCCGTCAAA-3’. Lipofectamine 3000 transfection reagent (Invitrogen) was used for all necessary transfections and transfections were performed strictly according to the manufacturer's instructions.

### Cell counting kit-8 (CCK-8) assay

The CCK-8 assay was used to determine cell activity. Cells were inoculated at a concentration of 3 × 10^3^ cells per well in a 96-well plate and incubated overnight with different concentrations of cisplatin for 2 days. The optical density was measured at 450 nm using a microplate reader. The IC50 of HeLa and Hela-CR cells were calculated by GraphPad software according to CCK-8 results.

### Quantitative real-time PCR (qRT-PCR)

Total RNA was extracted using RNAiso Plus (9108, Takara, Japan). mRNA was reverse transcribed into complementary cDNA using PrimeScript RT reagent Kit (RR047A, Takara, Japan). LncRNA was reverse transcribed into complementary cDNA using LnRcute lncRNA cDNA Strand Synthesis Kit (KR202, TIANGEN, China). miRNA was reverse transcribed into complementary cDNA using miRNA 1st Strand cDNA Synthesis Kit (MR201, Vazyme, China). Before reverse transcription, the whole genome DNA was removed according to the kit instructions. The relative expression of MEG3, miR-21 and PTEN were detected by TB Green® Premix Ex Taq™ II (RR820Q, Takara, Japan) to detect the level of MEG3, miR-21 and PTEN mRNAs. GAPDH or U6 was used as an internal control. Relative expression levels were calculated using the 2^−ΔΔCt^ method. Primers are shown in Table 1.

**Table 1:** Primers for quantitative real-time PCR

Gene name	Primer sequences
MEG3	Forward: 5′- TCCATGCTGAGCTGCTGCCAAG -3′ Reverse: 5′- AGTCGACAAAGACTGACACCC -3′
PTEN	Forward: 5′- CCCAGTCAGAGGCGCTATG -3′ Reverse: 5′- GGCAGACCACAAACTGAGGATT -3′
GAPDH	Forward: 5′- AATGGACAACTGGTCGTGGAC -3′ Reverse: 5′- CCCTCCAGGGGATCTGTTTG -3′
miR-21	Forward: 5′- GCACCTAGCTTATCAGACTGA -3′ Reverse: 5′- GTGCAGGGTCCGAGGT -3′
U6	Forward: 5′- GCTTCGGCAGCATATACTAAAAT -3′ Reverse: 5′- CGCTTCACGAATTTGCGTGTCAT -3′

### Colony formation assay

The cells were inoculated at a density of 500 cells/dish into 6-well plates and cultured in medium containing 10% fetal bovine serum, which was renewed every 3 days. Cells were cultured at 37 °C in a 5% CO2 incubator for approximately 2 weeks. The medium was washed off with PBS, fixed in 4% paraformaldehyde for 15 min, stained with 0.1% crystal violet for 30 min, photographed and counted using Image J software.

### Flow cytometry

Apoptosis was detected by flow cytometry. The treated cells were collected from each group and used to detect apoptosis according to the instructions of PE Annexin V Apoptosis Detection Kit (BD Pharmingen, USA). Apoptosis was also detected using a flow cytometer (EPICS, XL-4, Beckman, USA).

### Western blot analysis

Total cell protein was extracted by RIPA Lysis Buffer (P0013B, Beyotime, China). Protein concentrations were measured by BCA Protein Assay Kit (P0010, Beyotime, China). Protein extracts (30 μg) were loaded and separated by SDS–polyacrylamide gel and transferred to PVDF membranes. According to the molecular weight of the target protein, the PVDF membrane was cut along the marker and incubated with primary antibodies. Primary antibodies specific against Bax (1:2000, ab32503, Abcam, USA), cleaved-caspase 3 (1:500, ab32042, Abcam, USA), Bcl-2 (1:1000, ab32124, Abcam, USA), PTEN (1:1000, ab267787, Abcam, USA) and GAPDH (1:10,000, ab8245, Abcam, USA) were used to incubate membranes overnight at 4 °C. HRP-conjugated Goat Anti-Rabbit (1:10,000, ab6721, Abcam, USA) or HRP-conjugated Goat Anti-Mouse (1:10,000, ab6789, Abcam, USA) secondary antibodies were used for 1 h at room temperature. The signal was visualized using BeyoECL Plus (P0018S, Beyotime, China) and a gel imaging system, and Image J was used to calculate the gray values of the images.

### Cell migration assay

The cell migration was detected by transwell assay. 2 × 10^4^ number of cells were transferred to the top chamber of a noncoated membrane chamber in DMEM medium containing 5% fetal calf serum. DMEM containing 20% fetal calf serum was added to the lower chamber to function as a chemoattractant. After incubation for 24 h, nonmigrating cells were removed from the upper well, and the remaining migration cells were fixed with 4% paraformaldehyde and stained with crystal violet. The results were observed and photographed using a light microscope.

### Dual luciferase reporter assay

Use ENCORI (https://rna.sysu.edu.cn/encori/index.php) target prediction tool to prediction potential binding sites between miR-21 and PTEN or MEG3. A wild-type (MEG3 3' UTR-WT, PTEN 3' UTR-WT) and mutant (MEG3 3' UTR-MUT, PTEN 3' UTR-MUT) luciferase reporter gene plasmid containing the binding site were purchased from GenePharma (Shanghai, China). After cell plating, cell density was reached to approximately 50% and the WT or MUT luciferase plasmids and renilla luciferase plasmid were co-transfected with miR-21 mimic or miR NC in Hela-CR cells. 24 h after transfection, Luciferase activity was measured using the Dual-Glo Luciferase Assay System (E2920, Promega, USA) and GloMax® 20/20 Luminometer (E5311, Promega, USA) to detect firefly and renilla luciferase activity.

### In vivo studies

Six-week-old, female, nude mice were purchased from Beijing Biocytogen Co., Ltd (Beijing, China). The procedures for the handling and care of the mice were approved by the Animal Experimentation Ethics Committee of China Medical University (IACUC. No.2019388). Hela cells with knocked down MEG3 and control and overexpressed MEG3 Hela-CR cells were prepared, 1 × 10^7^ cells of each group in Matrigel (BD Biosciences, USA) were injected into the right flanks of mice to form xenograft tumors. As the tumor volumes reached about 100 mm^3^, mice were daily injected intraperitoneally with cisplatin (25 mg/kg) for 7 days. Meanwhile, 10 nmol [15] antagomiR-21, antagomiR-NC, agomiR-21 or agomiR-NC were injected intratumorally in a multisite injection manner every 3 days for 2 weeks. The average volume of the tumor was measured three times every 3 days. Tumor volumes were calculated using the formula: (Long x Wide^2^)/2.

### HE staining

Paraffin-embedded tumor tissue was cut into 4 μm thick sections and dewaxed in dimethylbenzene, hydrated in gradient alcohol, washed in distilled water, then immersed in hematoxylin for 15 min, washed to remove excess stain, fractionated in 1% ethanol hydrochloride for 10 s and washed in running water for 15 min before the tissue was stained with 0.5% eosin for 5 min. Xylene was clear and gradient alcohol was dehydrated. Neutral gum was used to seal the slices, which were observed under light microscopy and photographed.

### Immunohistochemistry staining

After euthanasia of mice, tumors were excised from these mice, fixed in 4% paraformaldehyde, and embedded in paraffin. Tissue specimens were incubated with antibodies against PTEN and a biotin-conjugated secondary antibody and then incubated with an avidin–biotin–peroxidase complex. Visualization was performed using amino-ethyl carbazole chromogen. Slides were analyzed using the Olympus BX43 microscope system (Olympus, Japan).

### Statistical analysis

Results were expressed as mean ± standard deviation (SD) of three independent experiments unless otherwise specified. Data were analyzed using Graph Prism 8.2 software (GraphPad Prism, USA). Student's t-test were used to analyze differences between groups. One-way analysis of variance (ANOVA) with Tukey's multiple comparison post hoc test were used to compare in more than two groups, and p < 0.05 was considered as significant difference.

## Results

### The dysregulation of MEG3 is associated with cisplatin resistance of cervical cancer cells

**Fig. 1:**
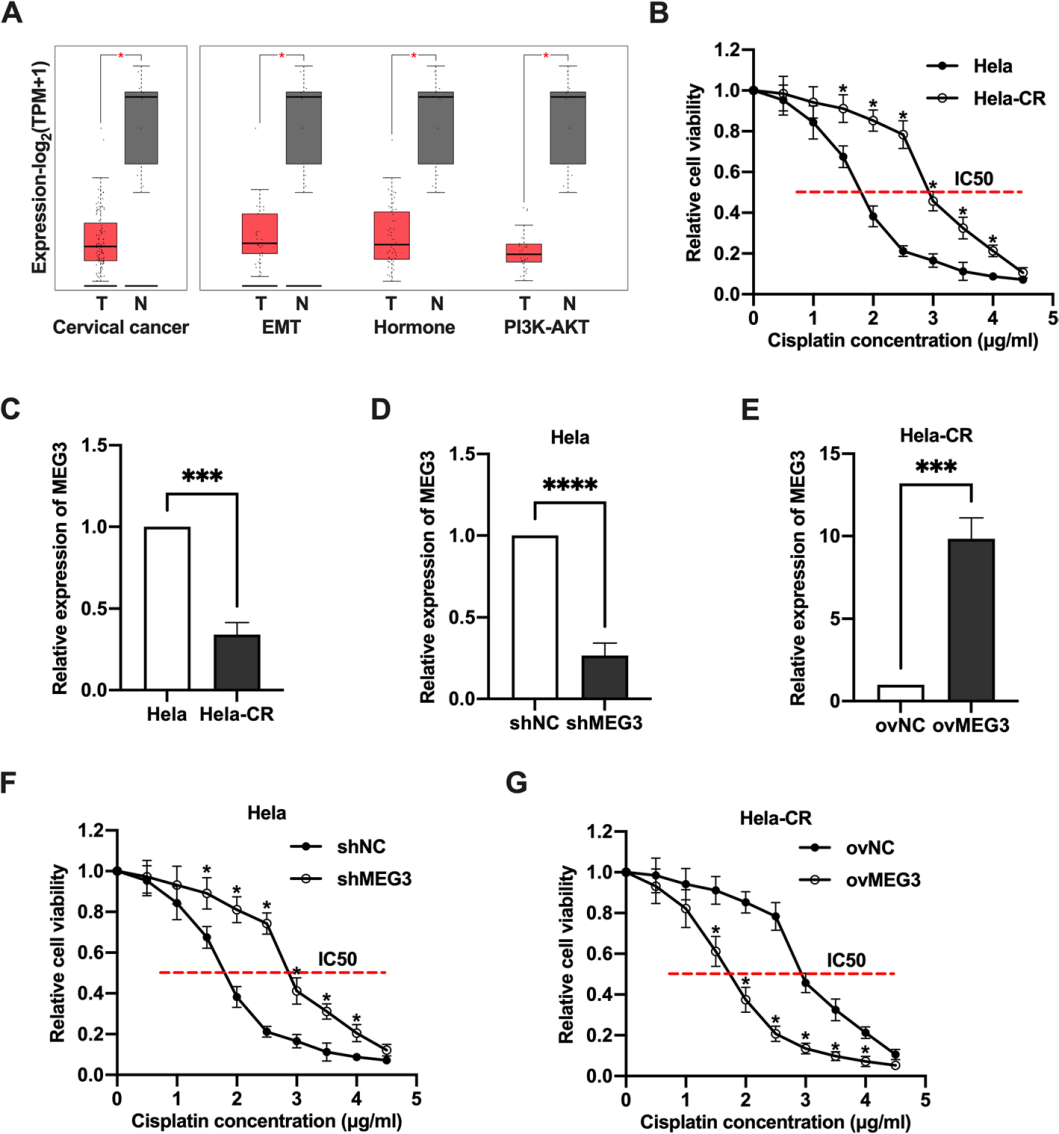
The dysregulation of MEG3 is associated with cisplatin resistance of cervical cancer cells. **A** MEG3 expression levels in cervical cancer samples from the TCGA database were analyzed by GEPIA (http://gepia2.cancer-pku.cn); **B** The IC50 values of Hela cells and Hela-CR cells by CCK-8 assay; **C**-**E** The expression levels of MEG3 in Hela cells and Hela-CR cells were measured by qRT-PCR; **F**-**G** The IC50 values of Hela cells and Hela-CR cells were then measured by CCK-8 assay. *indicate *P* < 0.05, ***indicate *P* < 0.001, ****indicate *P* < 0.0001

MEG3 expression levels in cervical cancer samples from the TCGA database were analyzed by GEPIA (http://gepia2.cancer-pku.cn). Obviously, MEG3 was downregulated in cervical cancer tissues compare with normal cervical tissues. In addition, compared with normal cervical tissues, the expression of MEG3 in EMT, Hormone, and PI3K-AKT subtypes of cervical cancer tissues was significantly lower (Fig. 1A). To investigate the effect of MEG3 on cisplatin resistance in cervical cancer cells, we established Hela-CR, which IC50 values were significantly higher (Fig. 1B) and the MEG3 levels were significantly lower than Hela cells (Fig. 1C). Next, Hela cells were transfected with lentivirus containing MEG3 knockdown to establish a Hela cell line with stable knockdown of MEG3 (Fig. 1D), and conversely, Hela-CR cells were transfected with MEG3 overexpression lentivirus to establish a Hela-CR cell line with stable high expression of MEG3 (Fig. 1E). As expected, knockdown of MEG3 increased the IC50 value of Hela cells (Fig. 1F) and overexpression of MEG3 decreased the IC50 value of Hela-CR cells (Fig. 1G). The above findings suggest that MEG3 is a potential biological marker of cisplatin resistance in cervical cancer.

### MEG3 affect the cisplatin resistance in cervical cancer cells

**Fig. 2:**
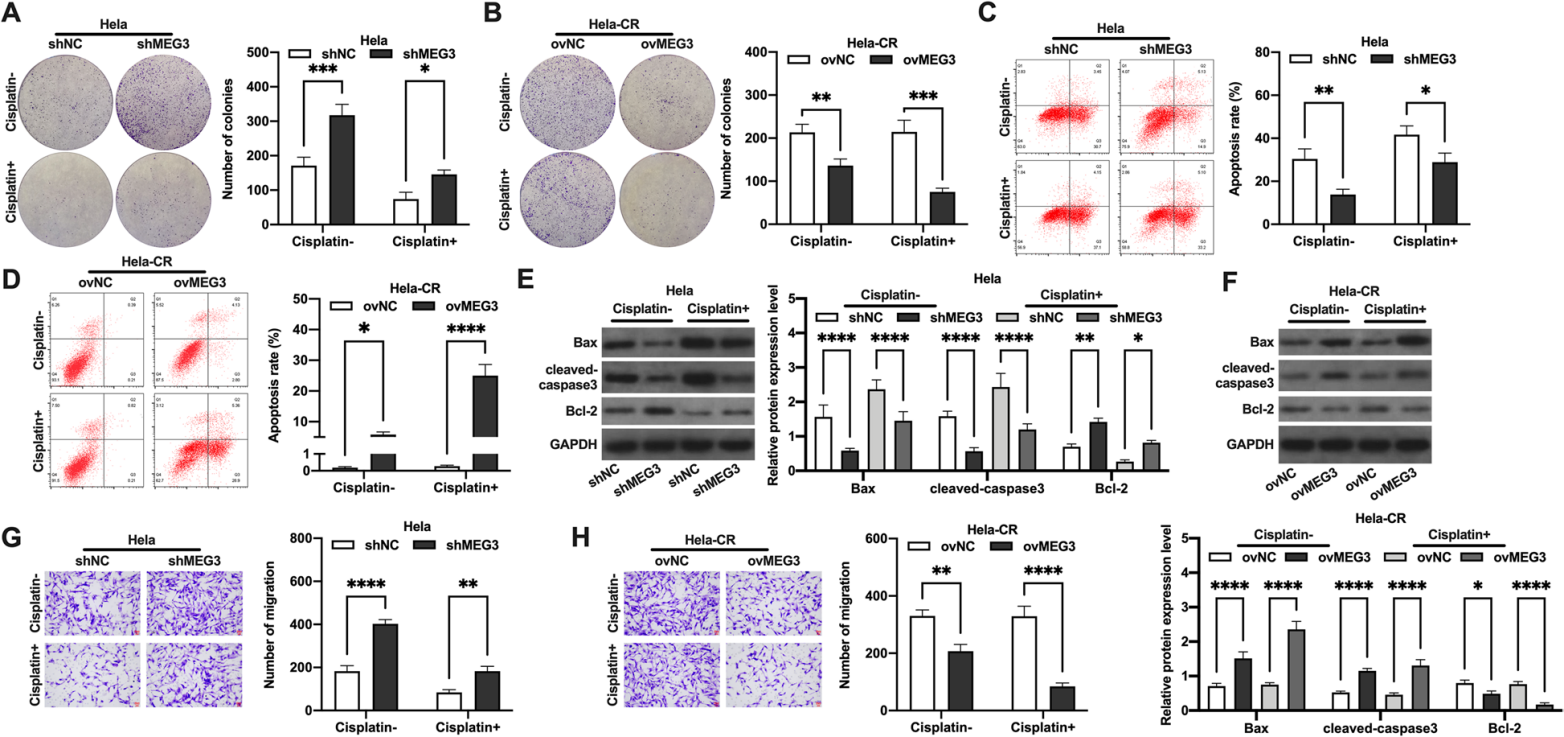
MEG3 affect the cisplatin resistance in cervical cancer cells. A-B. Colony formation assay; C-D Flow cytometry; E–F. The expression levels of apoptosis-related proteins by Western blot; G-H. Transwell assay. *indicate *P* < 0.05, **indicate *P* < 0.01, ***indicate *P* < 0.001, ****indicate *P* < 0.0001

In view of the potential oncogenic effect of MEG3, we examined the biological functions of Hela cells treated with 1 μg/ml of cisplatin and Hela-CR cells treated with 3 μg/ml of cisplatin under untreated or treated conditions. The results showed that cisplatin treatment inhibited the proliferation, migration ability and decreased the apoptosis rate of Hela cells, and knockdown of MEG3 reversed the effect of cisplatin (Fig. 2A, 2C, 2E and 2G). Overexpression of MEG3 inhibited the proliferation, migration ability and increased the apoptosis rate of Hela-CR cells, and the effect was more pronounced after cisplatin treatment (Fig. 2B, 2D, 2F and 2H). In summary, we reported that MEG3 could promote the sensitivity of CC cells to cisplatin.

### MEG3 acts as a sponge of miR-21

**Fig. 3:**
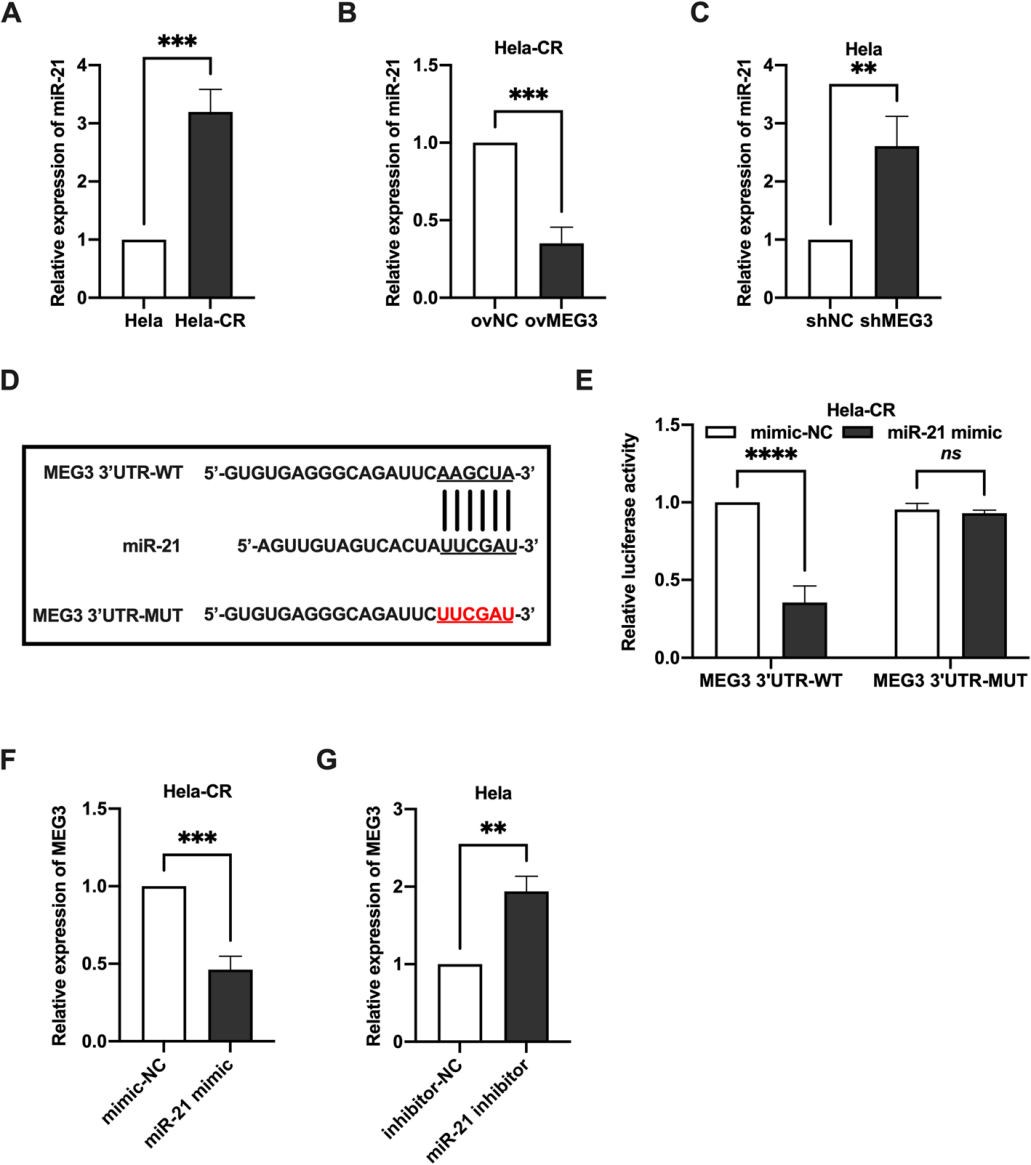
MEG3 acts as a sponge of miR-21. **A**-**C** The miR-21 expression levels in Hela cells and Hela-CR cells by qRT-PCR; **D** The potential binding sites between MEG3 and miR-21 were predicted by bioinformatics analysis; E. The results of the dual luciferase reporter gene assay; F-G. The MEG3 expression levels in Hela cells and Hela-CR cells by qRT-PCR. **indicate *P* < 0.01, ***indicate *P* < 0.001, ****indicate *P* < 0.0001

In a variety of cancers, lncRNAs act as ceRNA sponging miRNAs, increasing the expression of potential target mRNAs. We found that the miR-21 expression in Hela-CR cells was significantly higher than that in Hela cells (Fig. 3A), and overexpression of MEG3 decreased the miR-21 expression in Hela-CR cells (Fig. 3B), and conversely, knockdown of MEG3 increased miR-21 expression in Hela cells (Fig. 3C). The potential binding sites between MEG3 and miR-21 were predicted by bioinformatics analysis (Fig. 3D) and verified by dual luciferase reporter assay (Fig. 3E). The next test was whether miR-21 affected MEG3 expression. We found that transfection of miR-21 mimic decreased MEG3 expression in Hela-CR cells (Fig. 3F) and conversely, transfection of miR-21 inhibitor increased MEG3 expression in Hela cells (Fig. 3G). In conclusion, MEG3 acts as a sponge of miR-21.

### MEG3 positively regulates PTEN by sponging miR-21

**Fig. 4:**
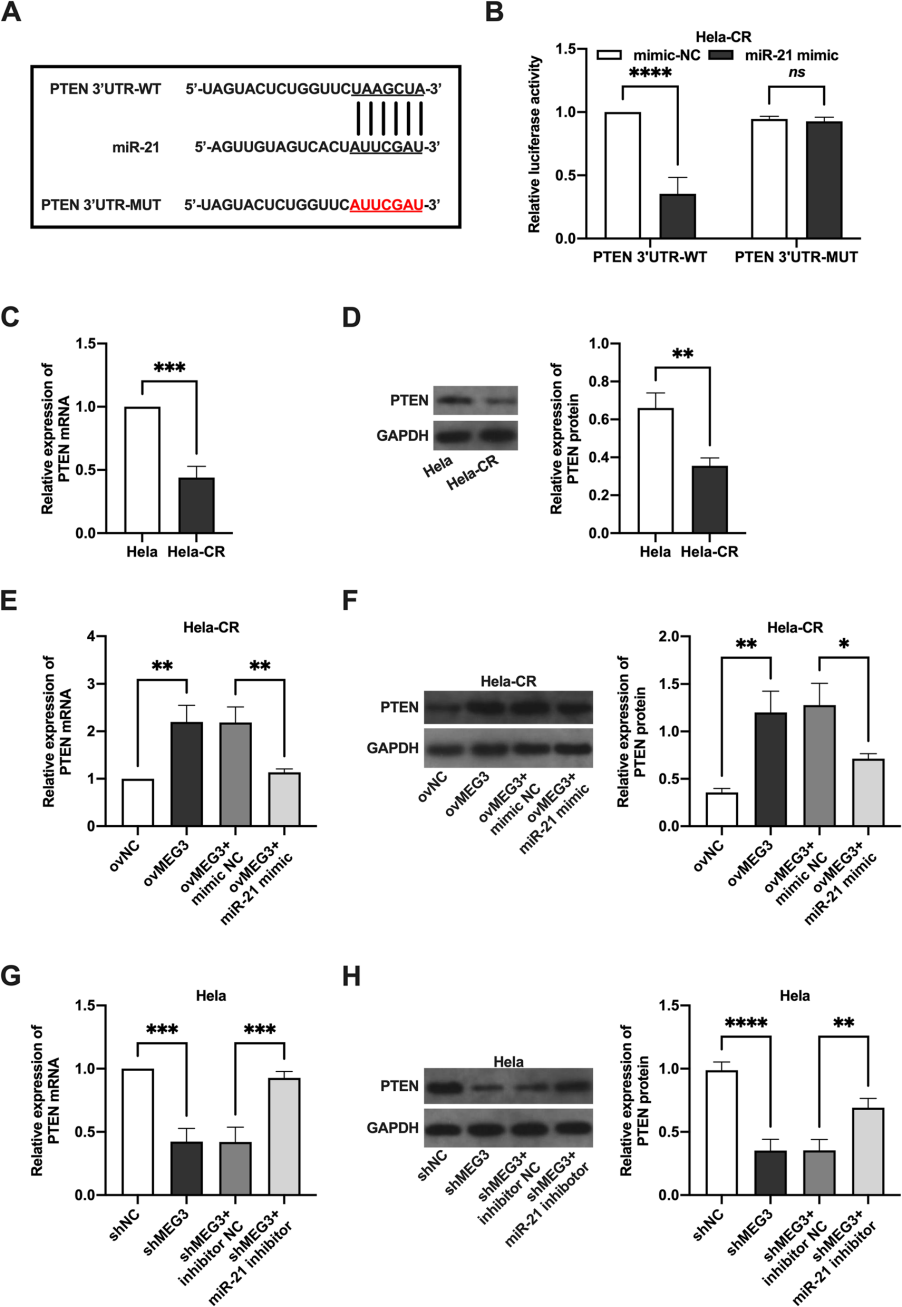
MEG3 positively regulates PTEN by sponging miR-21. **A** Potential binding sites between miR-21 and PTEN were predicted by bioinformatic analysis. **B** The results of the dual luciferase reporter gene assay; **C**, **E**, **G** The PTEN mRNA expression levels in Hela cells and Hela-CR cells by qRT-PCR assay; **D**, **F**, **H** The PTEN protein expression levels in Hela cells and Hela-CR cells by Western blot assay. *indicate *P* < 0.05, **indicate *P* < 0.01, ***indicate *P* < 0.001, ****indicate *P* < 0.0001

It has been reported that miR-21 can reduce cisplatin sensitivity in cervical cancer cells by targeting PTEN [16]. We hypothesized that the miR-21/PTEN axis could interact with MEG3 through a ceRNA pattern. Potential binding sites between miR-21 and PTEN were predicted by bioinformatic analysis (Fig. 4A) and verified by dual luciferase reporter assay (Fig. 4B). Next, the results showed that the expression levels of PTEN mRNA (Fig. 4C) and protein (Fig. 4D) were significantly lower in Hela-CR cells than in Hela cells. Overexpression of MEG3 increased PTEN mRNA (Fig. 4E) and protein (Fig. 4F) expression levels in Hela-CR cells, while transfection with miR-21 mimic reversed the effect of MEG3 overexpression. Conversely, knockdown of MEG3 decreased PTEN mRNA (Fig. 4G) and protein (Fig. 4H) expression levels in Hela cells, while transfection with miR-21 inhibitor reversed the effect of knockdown of MEG3. These results suggest that MEG3 positively regulates PTEN expression by sponging miR-21.

### The regulative role of MEG3/miR-21/PTEN axis on cisplatin resistance in cervical cancer cells

**Fig. 5:**
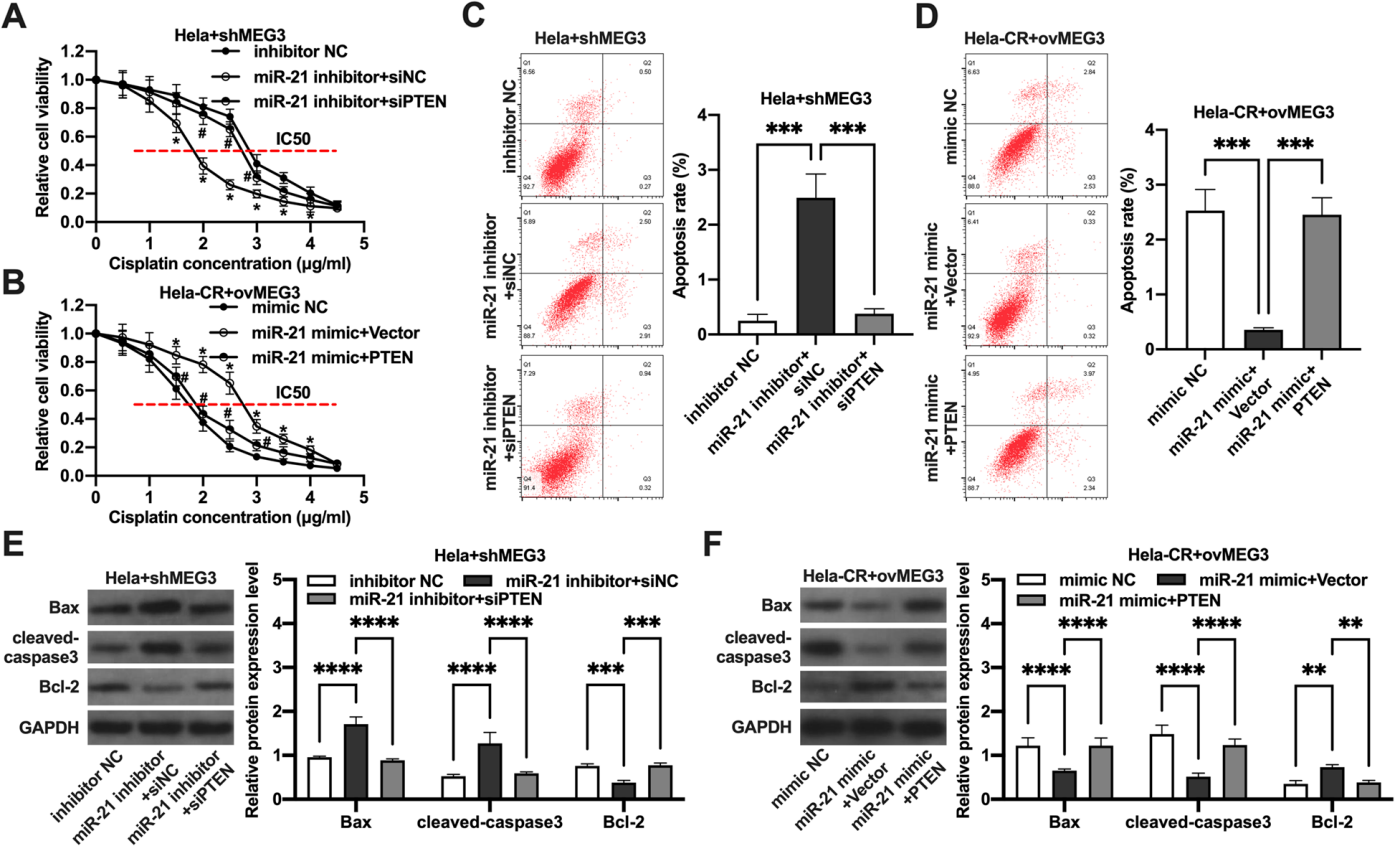
The regulative role of MEG3/miR-21/PTEN axis on cisplatin resistance in cervical cancer cells. **A**-**B** CCK-8 assays; **C**-**D** apoptosis detected by flow cytometry; **E**–**F** the expression levels of apoptosis-related proteins detected by Western blot. **indicate *P* < 0.01, ***indicate *P* < 0.001, ****indicate *P* < 0.0001

Further evidence of the function of the MEG3/miR-21/PTEN ceRNA pathway was demonstrated. Firstly, the miR-21 inhibitor reduced the IC50 values of Hela cells with knockdown of MEG3, while silencing PTEN reversed the effect of miR-21 inhibitor (Fig. 5A). Conversely, miR-21 mimic increased the IC50 values of Hela-CR cells overexpressing MEG3, while overexpression of PTEN reversed the effect of miR-21 mimic (Fig. 5B). Subsequently, the miR-21 inhibitor increased the apoptosis rate of Hela cells with knockdown of MEG3, while silencing PTEN reversed the effect of miR-21 inhibitor (Fig. 5C, E). Conversely, miR-21 mimic decreased the apoptosis rate of Hela-CR cells overexpressing MEG3, while overexpression of PTEN reversed the effect of miR-21 mimic (Fig. 5D, F). The above results indicated that MEG3 promoted the sensitivity of cervical cancer cells to cisplatin through the MEG3/miR-21/PTEN axis.

### Effect of the MEG3/miR-21/PTEN axis on cisplatin resistance in cervical cancer cells in vivo

To investigate the role of MEG3/miR-21/PTEN axis in cisplatin resistance in CC in vivo, the xenograft tumor model of nude mice was established. After cisplatin treatment, the tumor volume, weight and cell necrosis of Hela cells containing knockdown of MEG3 were significantly increased compared to Hela cell, and the administration of antagomiR-21 reversed the effect of knockdown of MEG3 (Fig. 7A-D). Conversely, the tumor volume, weight and cell necrosis of Hela-CR cells overexpressing MEG3 was significantly reduced after cisplatin treatment compared to those Hela-CR cells without MEG3 overexpressed, and the effect of MEG3 overexpression was reversed by the administration of agomiR-21 (Fig. 7A-D).

**Fig. 6:**
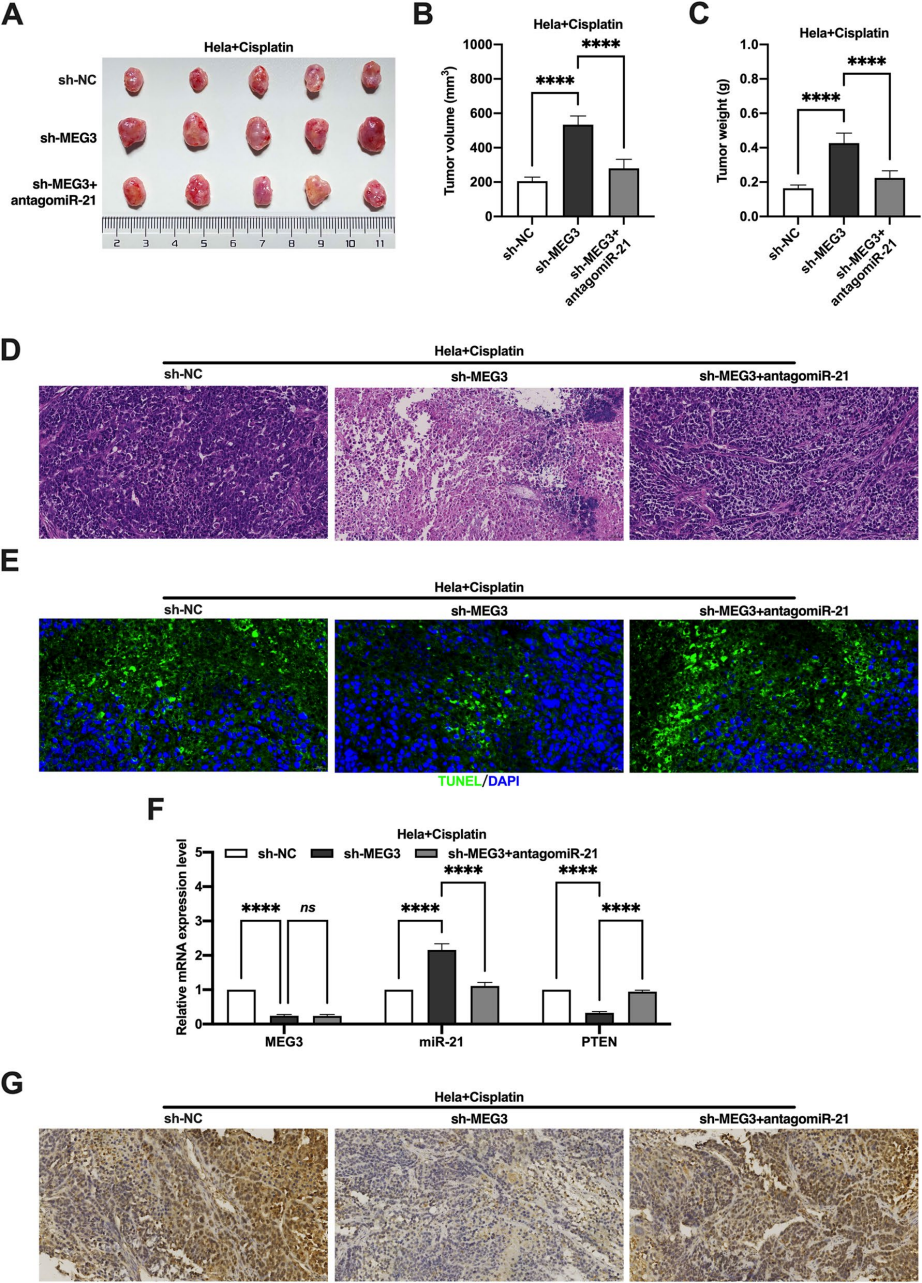
Effect of the MEG3/miR-21/PTEN axis on cisplatin resistance in cervical cancer cells in vivo. **A** The xenograft tumor model of nude mice; **B** The tumor volume; **C** The tumor weight; **D** HE staining; **E** TUNEL assay; **F** qRT-PCR was performed to detect the expression levels of MEG3, miR-21 and PTEN mRNA in xenograft tumor; **G** Immunohistochemistry detected the expression levels of PTEN. ****indicate *P* < 0.0001

**Fig. 7:**
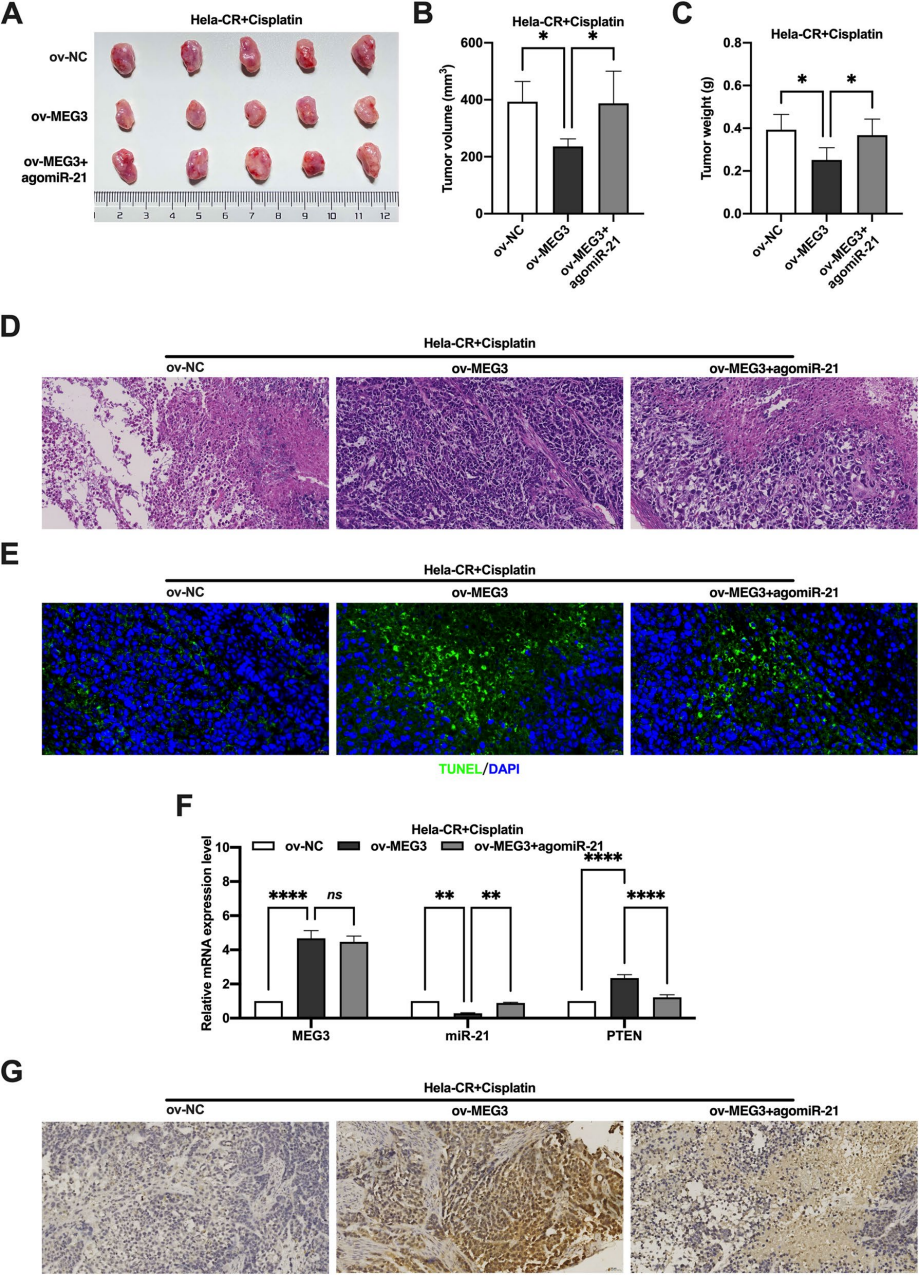
Effect of the MEG3/miR-21/PTEN axis on cisplatin resistance in cervical cancer cells in vivo. **A** The xenograft tumor model of nude mice; **B** The tumor volume; **C** The tumor weight; **D** HE staining; **E** TUNEL assay; F qRT-PCR was performed to detect the expression levels of MEG3, miR-21 and PTEN mRNA in xenograft tumor; **G** Immunohistochemistry detected the expression levels of PTEN. *indicate *P* < 0.05, **indicate *P* < 0.01, ****indicate *P* < 0.0001

Furthermore, the results of TUNEL assay showed that the level of apoptosis in mice with Hela cells knockdown MEG3 was reduced after cisplatin treatment, and the administration of antagomiR-21 reversed the effect of knockdown MEG3 (Fig. 6E). Conversely, apoptosis was significantly increased in Hela-CR cells overexpressing MEG3 after cisplatin treatment, and administration of agomiR-21 reversed the effect of overexpression of MEG3 (Fig. 7E). The results also showed that knockdown of MEG3 decreased PTEN expression, and administration of antagomiR-21 increased PTEN expression in Hela cell xenograft (Fig. 6F-G). Conversely, overexpression of MEG3 increased PTEN expression, and administration of agomiR-21 decreased the PTEN expression in Hela-CR cell xenograft (Fig. 7F-G). The above results confirmed that MEG3 promotes the sensitivity of CC cells to cisplatin through the MEG3/miR-21/PTEN axis in vivo.

## Discussion

As an important gynecological malignancy, cervical cancer has a high mortality rate and a poor prognosis. Although various chemotherapeutic agents have been developed for the treatment of cervical cancer, resistance to these agents is an important factor in the poor prognosis of patients with cervical cancer. Cisplatin is a common compound and chemotherapy with paclitaxel in combination with cisplatin is the first-line chemotherapy regimen for cervical adenocarcinoma. However, as the tumour progresses, the cancer cells become less sensitive to cisplatin and thus develop resistance to cisplatin. Therefore, there is an urgent need to investigate the specific molecular mechanisms that promote cisplatin resistance.

Both inhibition of apoptosis and promotion of cell proliferation have been suggested as possible mechanisms of cisplatin resistance in cancer cells. Many evidence suggests that lncRNAs affect cisplatin resistance in cancer cells by inhibiting apoptosis and promoting cell proliferation [17–19]. In this study, we analyzed MEG3 expression levels in cervical cancer from the TCGA database by using the GEPIA visual online analysis tool, and the results showed that MEG3 expression levels were significantly lower in cervical cancer than in normal tissues. Moreover, MEG3 expression was significantly lower in cervical adenocarcinoma cisplatin-resistant cell lines. Functional analysis experiments indicated that MEG3 promoted the sensitivity of cervical adenocarcinoma cells and drug-resistant cells to cisplatin.

LncRNAs have been widely reported to sponge with miRNAs and thus promote cisplatin resistance in various cancers, for example, lncRNA FGD5-AS1 enhances cisplatin resistance in lung adenocarcinoma by suppressing miR-142 expression [18]. miR-21, a microRNA, is expressed at high levels in a variety of solid tumors and affects biological functions such as adhesion, metastasis, and invasion of tumor cells [20]. miR-21 has been found to play a key regulatory role in drug resistance in a variety of tumors, for example, inhibition of miR-21 in hepatocellular carcinoma cells resulted in reduced cell growth, metastasis, invasiveness, and drug resistance [21, 22]. In contrast, transfection of normal tumor cells with miR-21 resulted in a substantial increase in cell metastasis accompanied by increased drug resistance [23]. The above reports suggest that miR-21 plays an important regulatory role in tumor chemoresistance. Several studies have shown that miR-21 can modulate the expression of PTEN by binding to its 3'UTR, thereby regulating its tumor resistance. For example, miR-21 reduced the sensitivity of K562 cell line to adriamycin by inhibiting PTEN expression [24]. The anti-tumor effect of curcumin analogue CDF in gemcitabine-resistant pancreatic cancer cells was mainly achieved by down-regulating miR-21 and thus up-regulating PTEN expression [25]. In this study, we verified through a dual luciferase reporter gene assay the binding of MEG3, PTEN and miR-21 and the expression of MEG3 and PTEN were negatively regulated by miR-21. Furthermore, the different assays showed that MEG3 promoted cisplatin sensitivity in CC cells by sponging miR-21 and increasing PTEN expression. This result was confirmed in vivo using xenograft tumor model of nude mice.

## Conclusions

In conclusion, this study shows that MEG3 enhances cisplatin sensitivity in cervical cancer cells by regulating the miR-21/PTEN axis, and that MEG3 acts as a ceRNA of the miR-21/PTEN axis to exert oncogenic effects and influence cisplatin resistance in cervical cancer. This study may contribute to reduce chemoresistance in cervical cancer and providing new therapeutic targets for cervical cancer.

## Supplementary Information

**Additional** **file** **1.**

## Data Availability

The datasets generated and/or analysed during the current study are available in the GEPIA (http://gepia2.cancer-pku.cn) repository.

## Abbreviations

ANOVA: One-way analysis of variance
CC: Cervical cancer
CCK-8: Cell counting kit-8
DLK1: Delta-like 1 homologue
Hela-CR: Cisplatin-resistant Hela cell lines
lncRNA: Long non-coding RNA
MEG3: Human maternally expressed gene 3
qRT-PCR: Quantitative real-time polymerase chain reaction
SD: Standard deviation
