# Supplementary material for: LncRNA MEG3 promotes cisplatin sensitivity of cervical cancer cells by regulating the miR-21/PTEN axis
Source: BMC Cancer. 2022 Nov 7;22:1145. doi: 10.1186/s12885-022-10188-0 (PMC9641762; doi:10.1186/s12885-022-10188-0)

Figure 2E

BAX


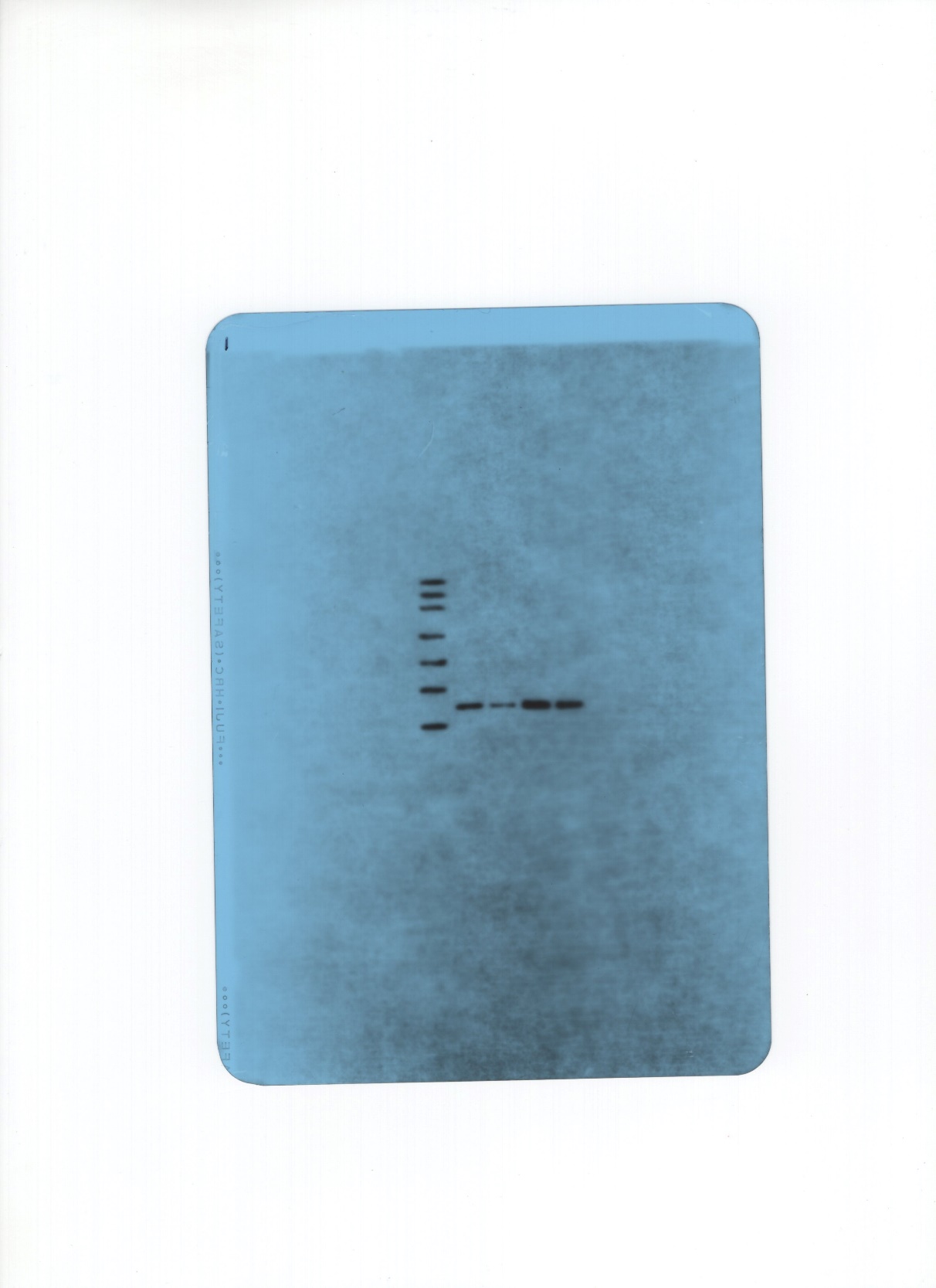


Bcl-2


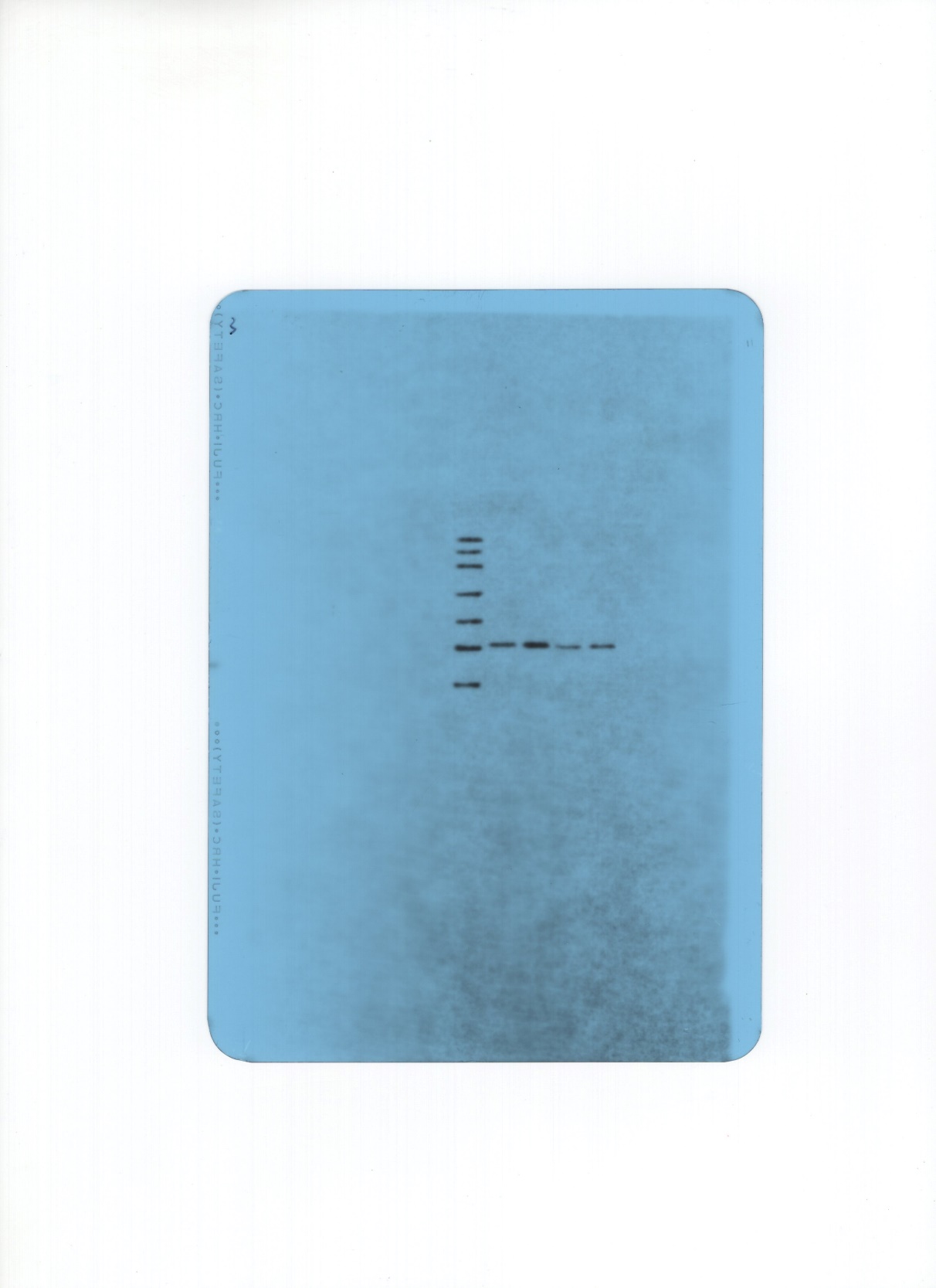


cleaved-caspase 3


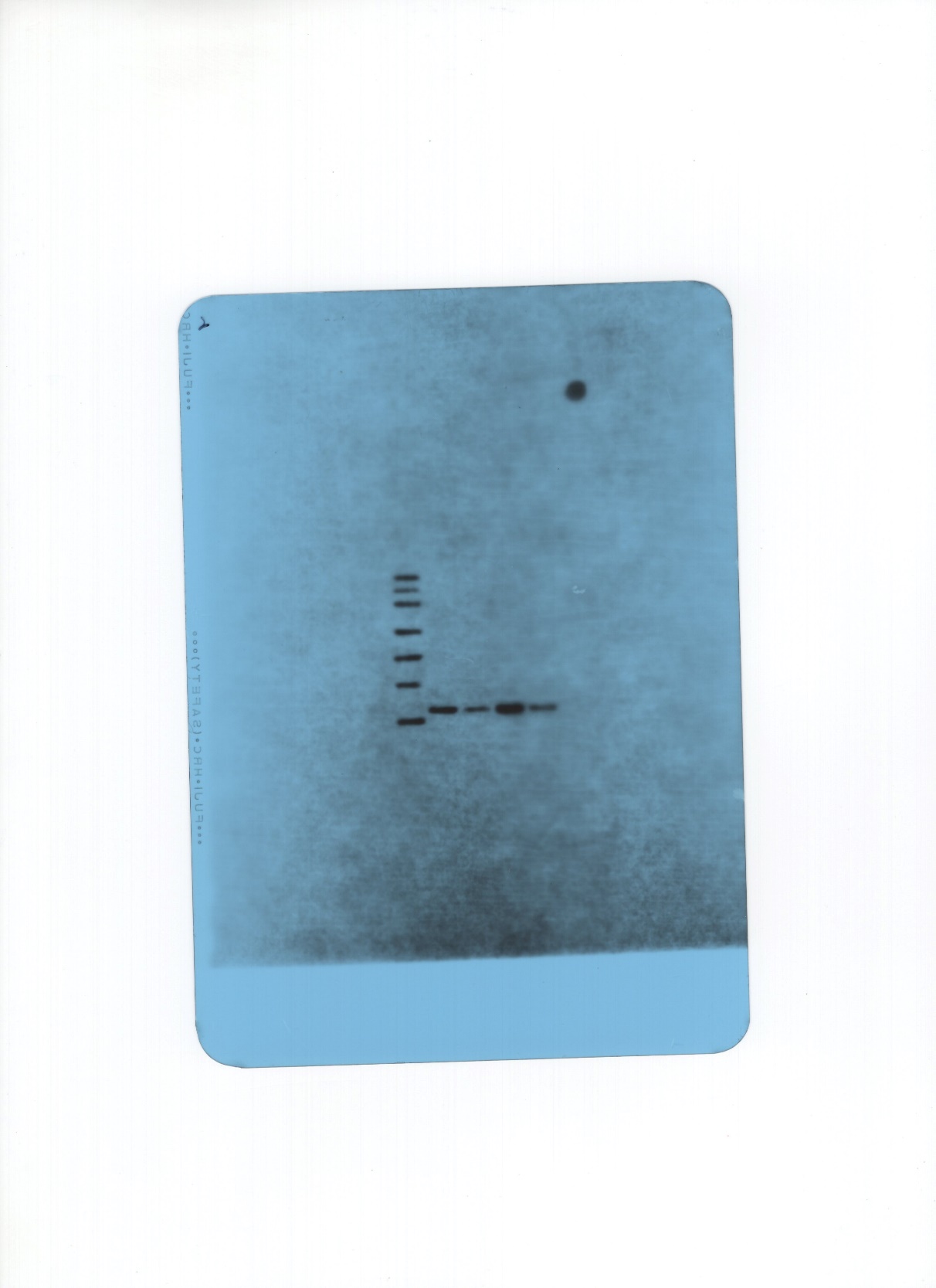


GAPDH


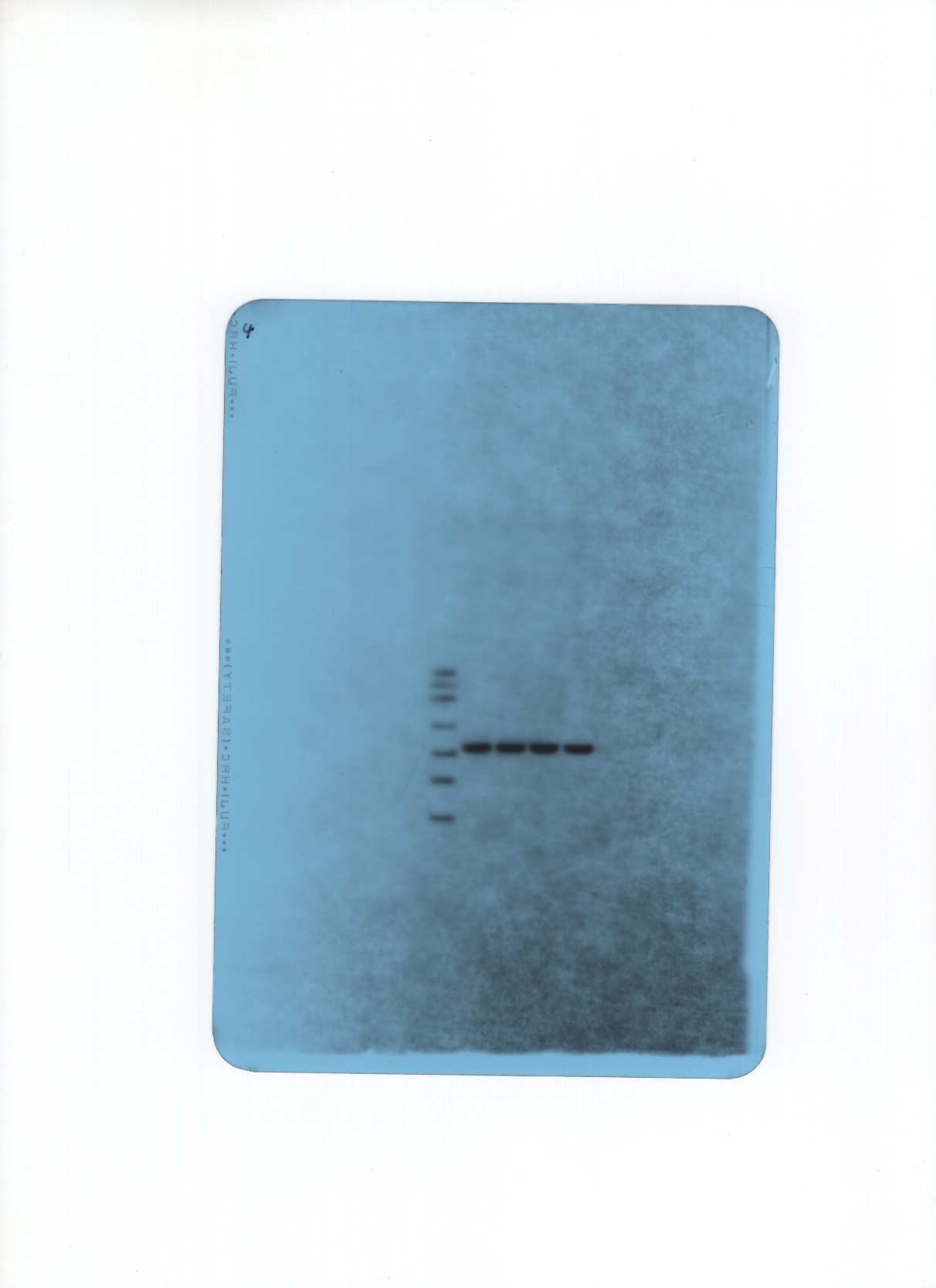


Figure 2F

BAX


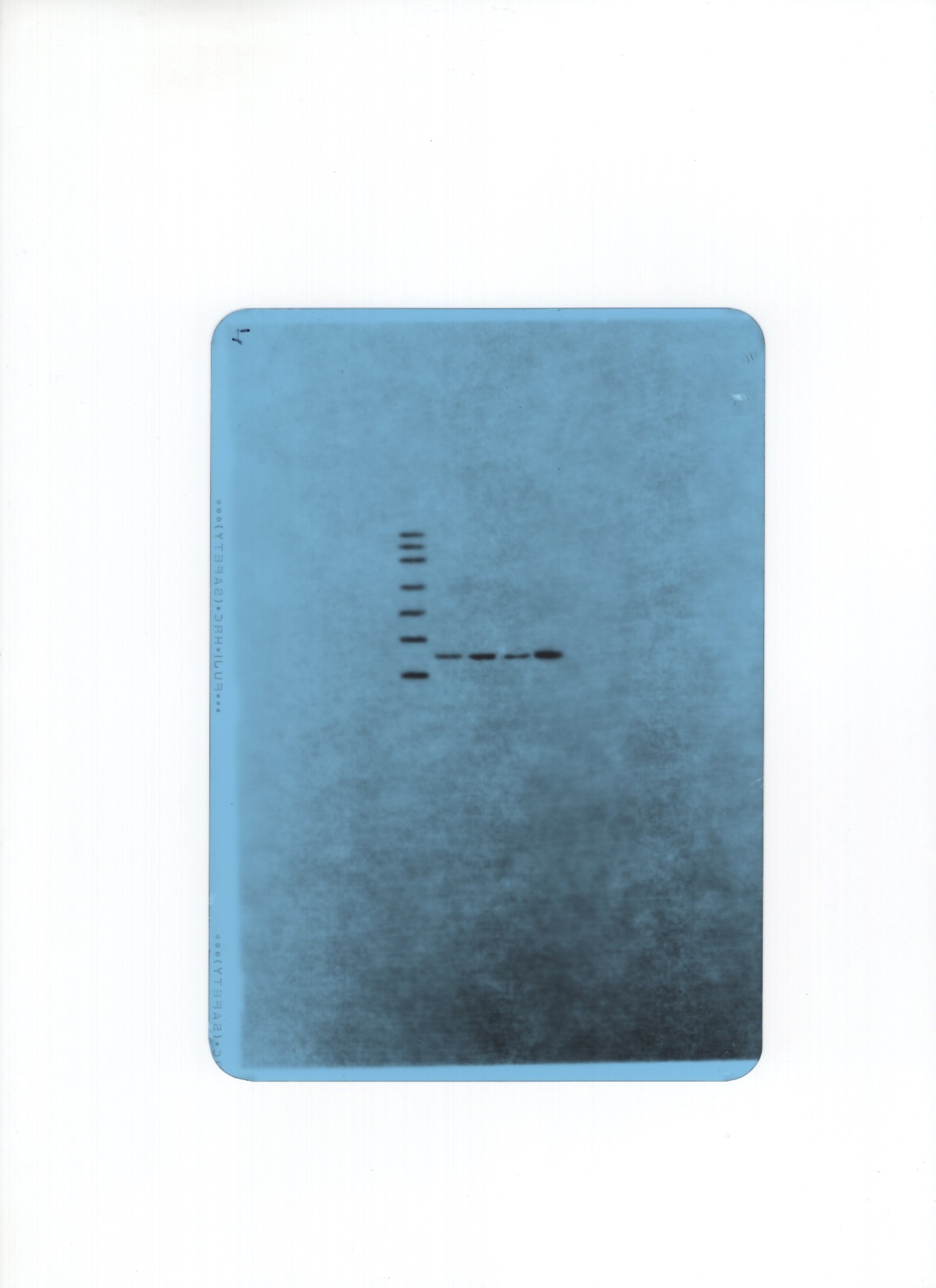


Bcl-2


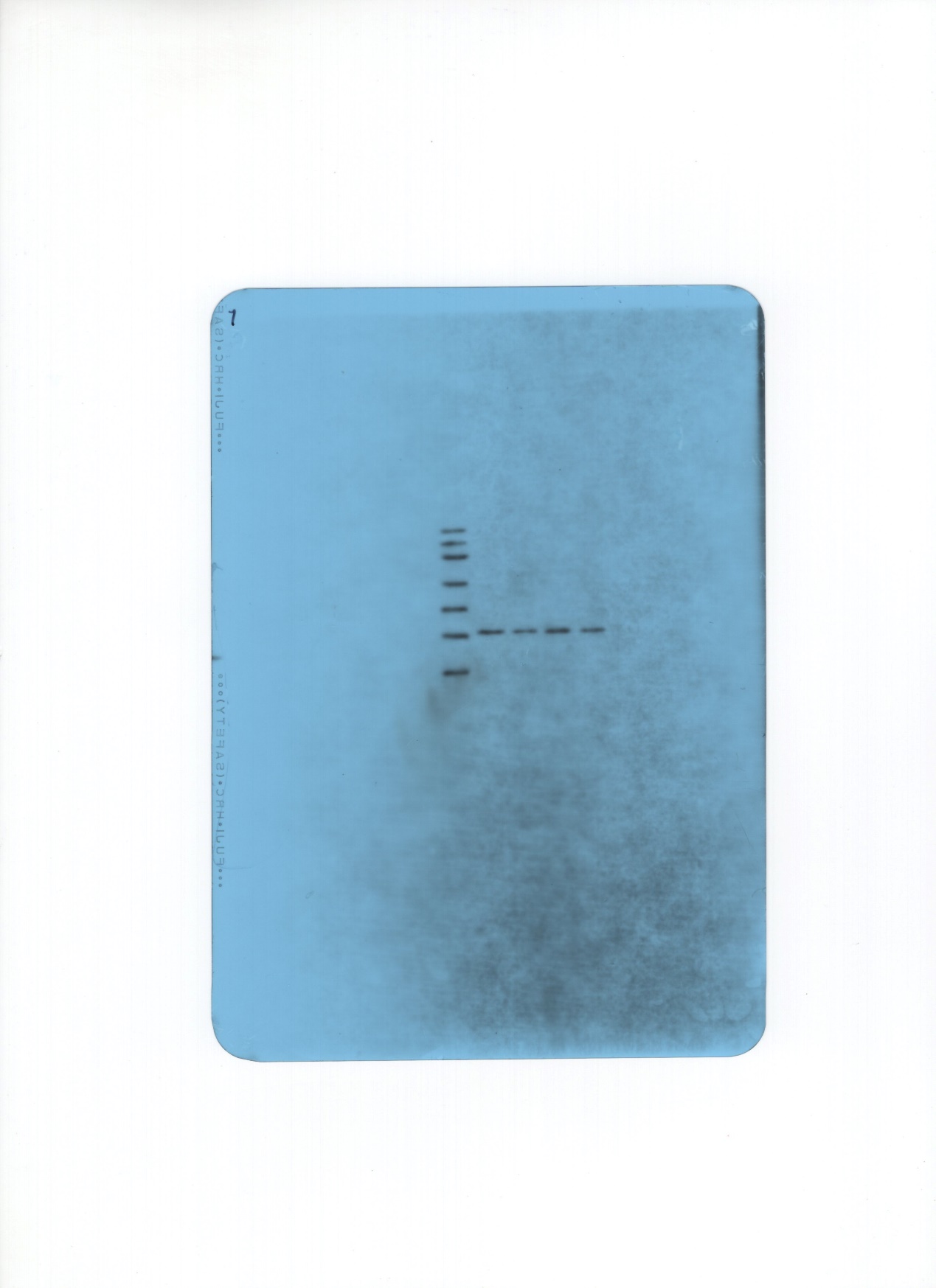


cleaved-caspase 3


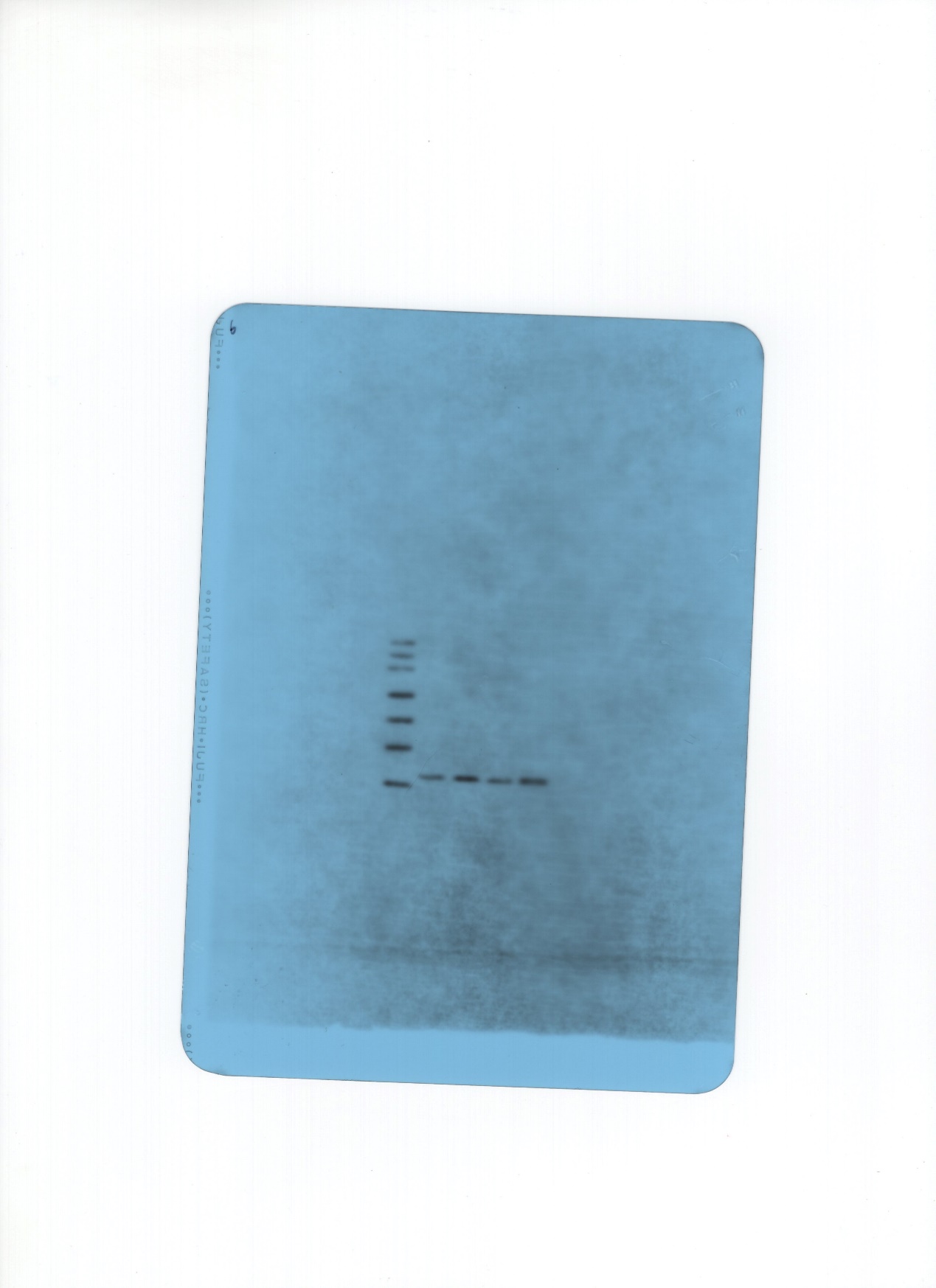


GAPDH


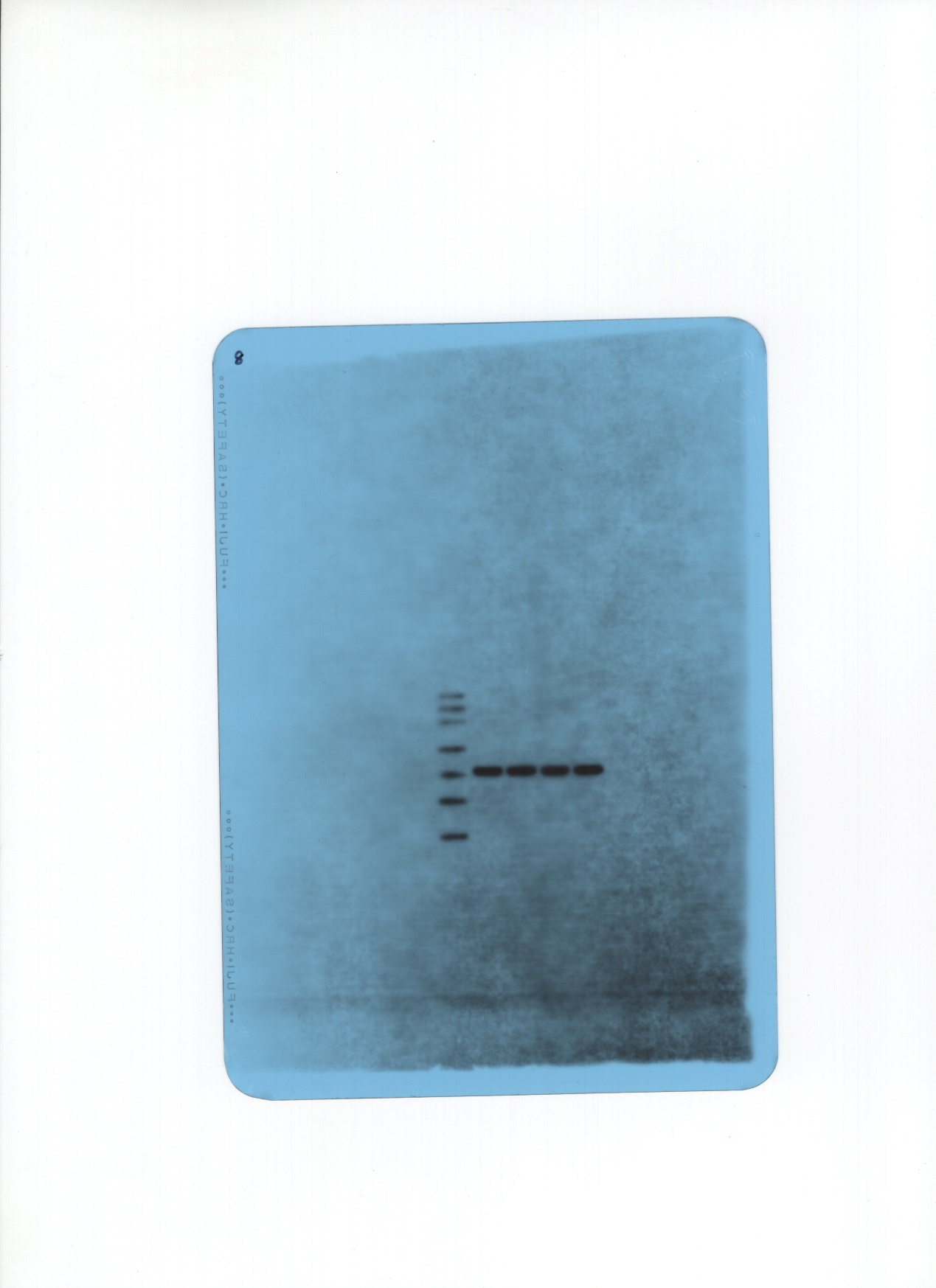


Figure 4D

PTEN


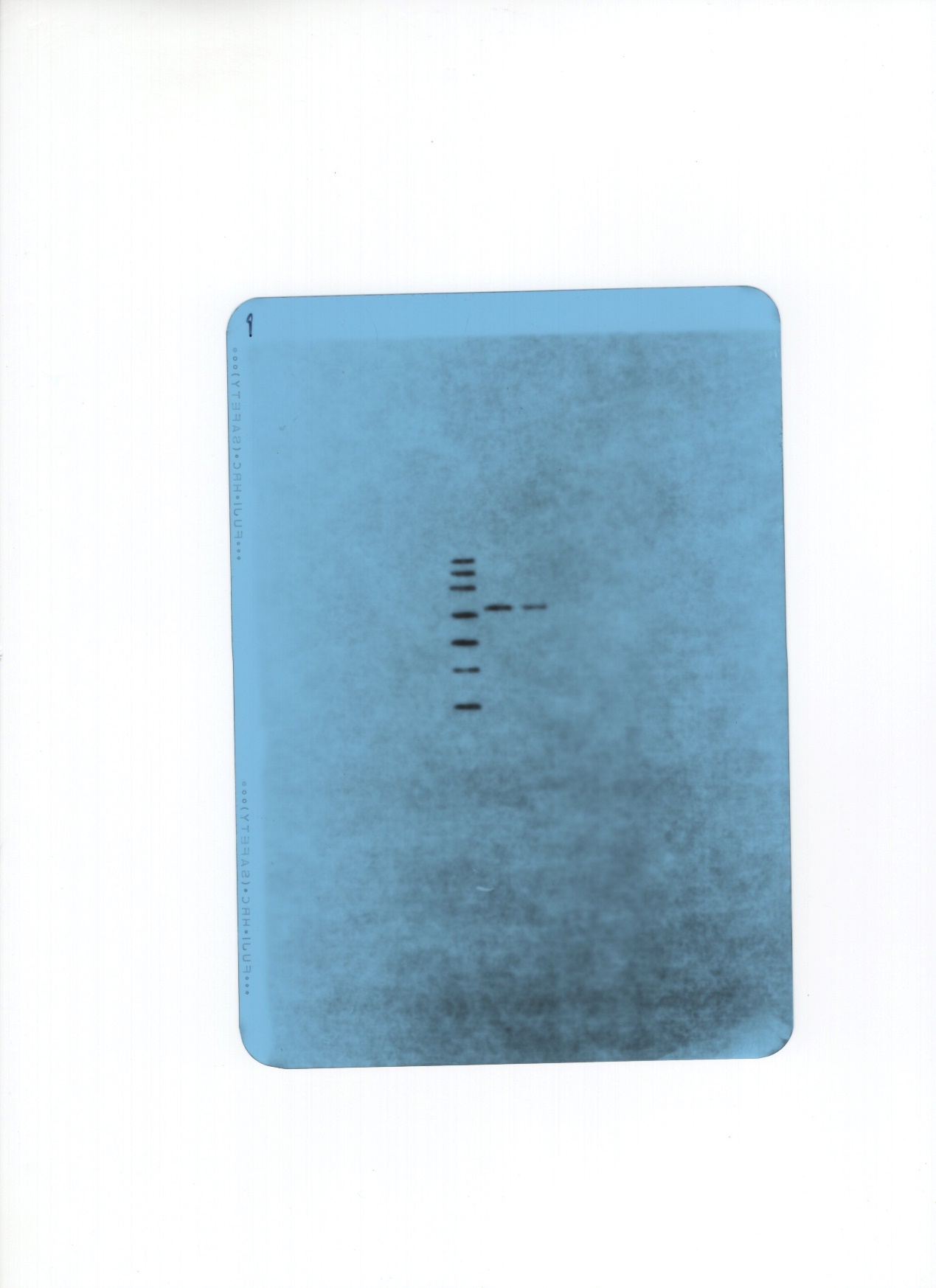


GAPDH


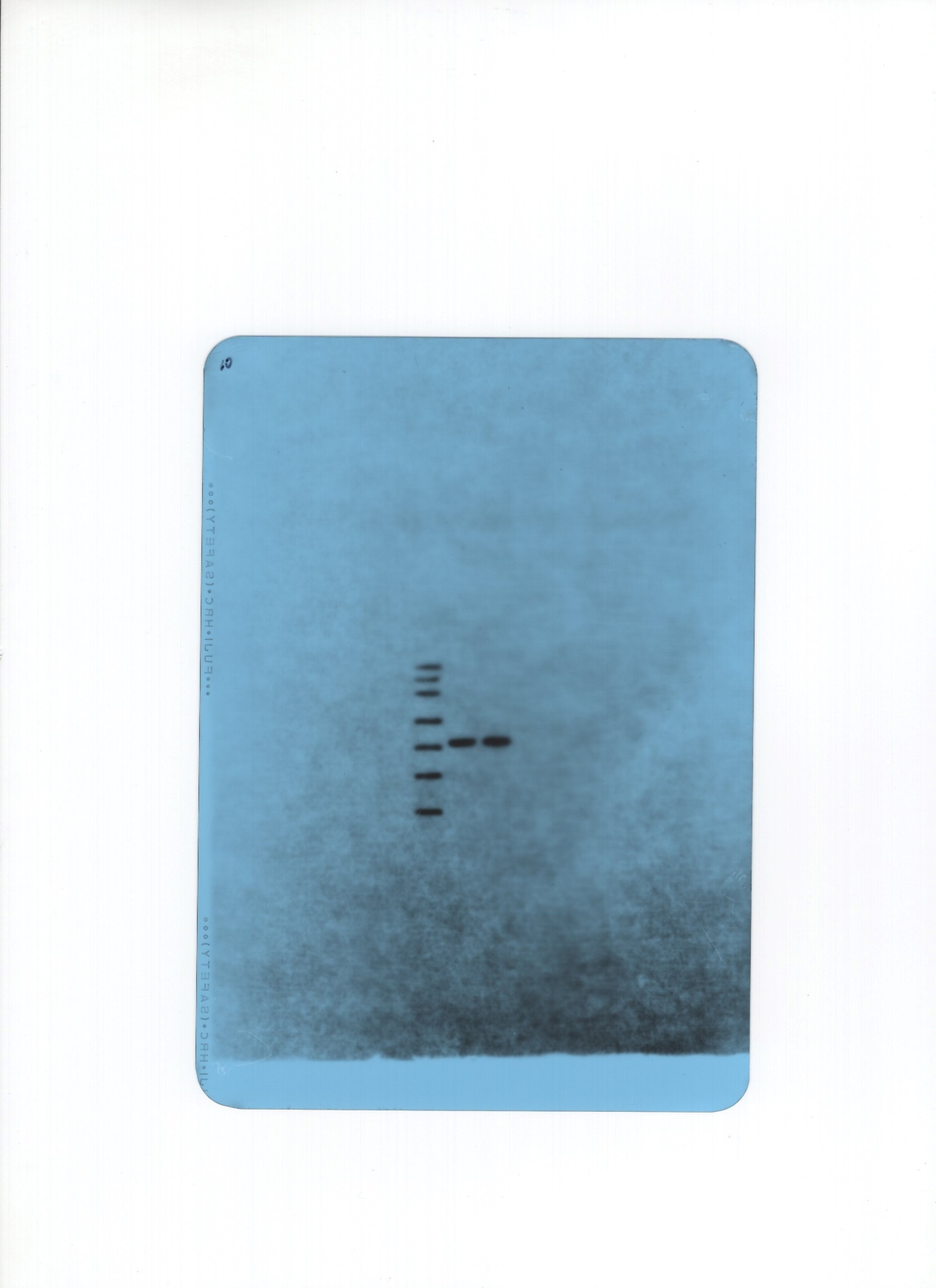


Figure 4F

PTEN


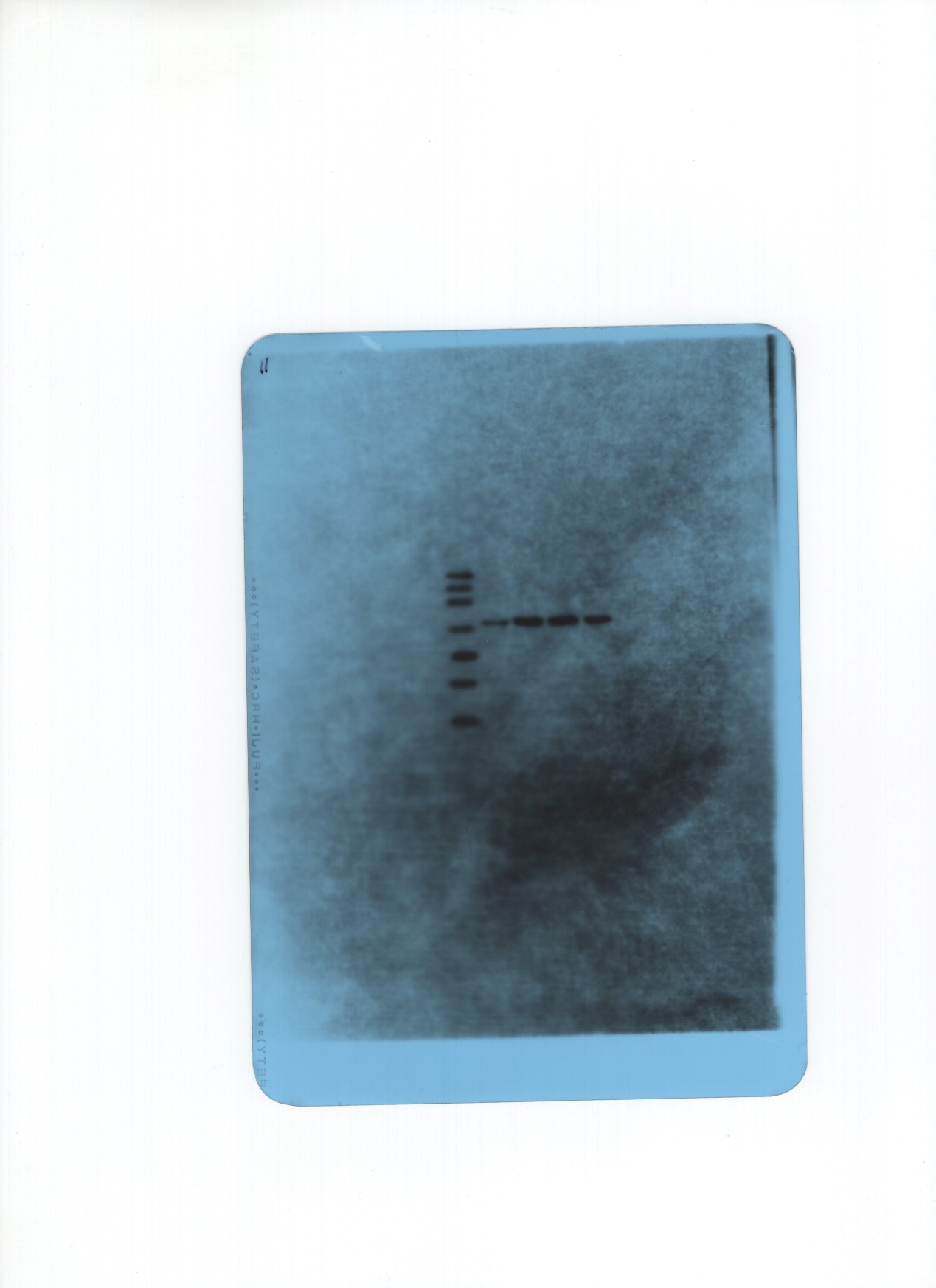


GAPDH


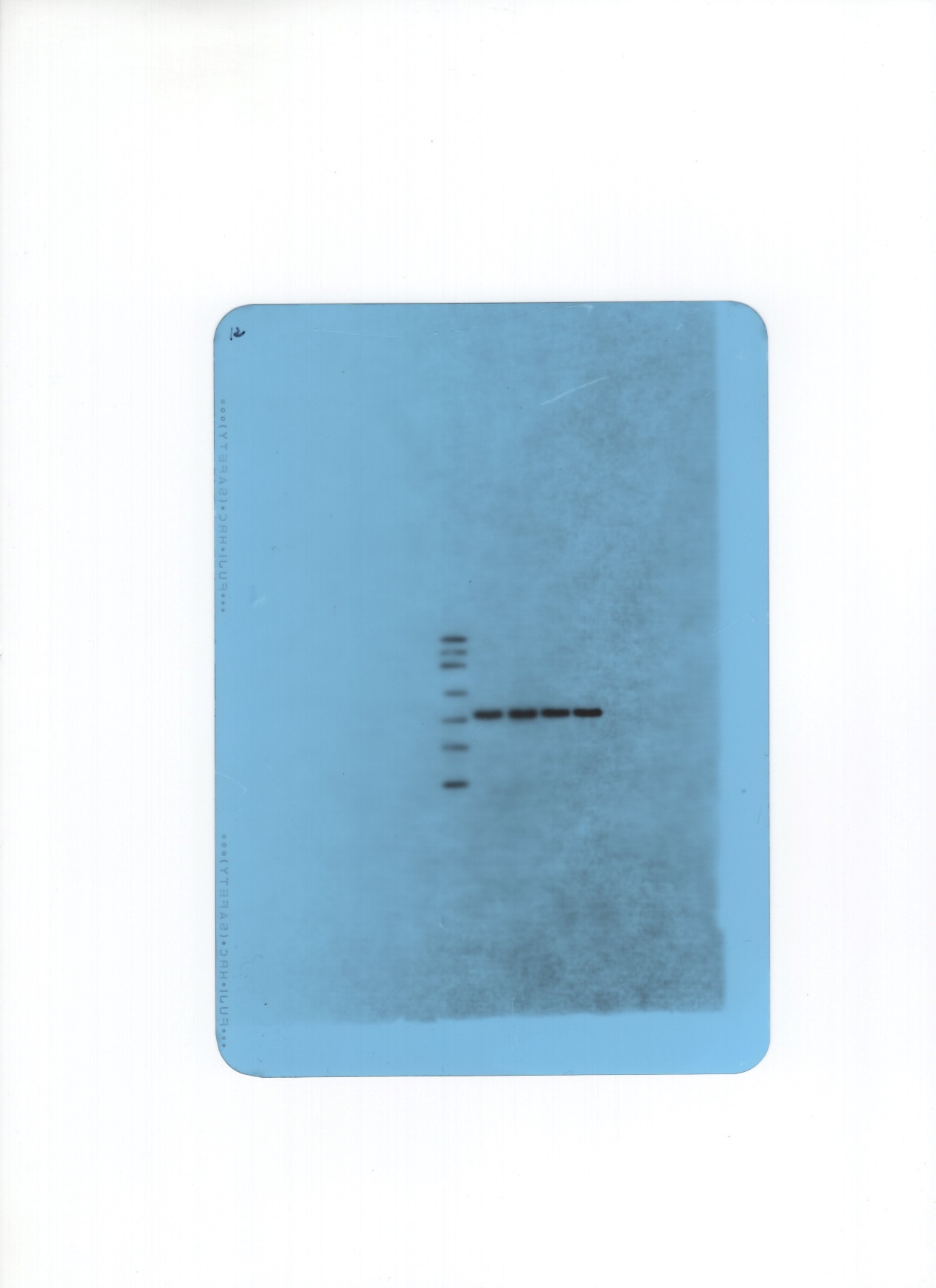


Figure 4H

PTEN


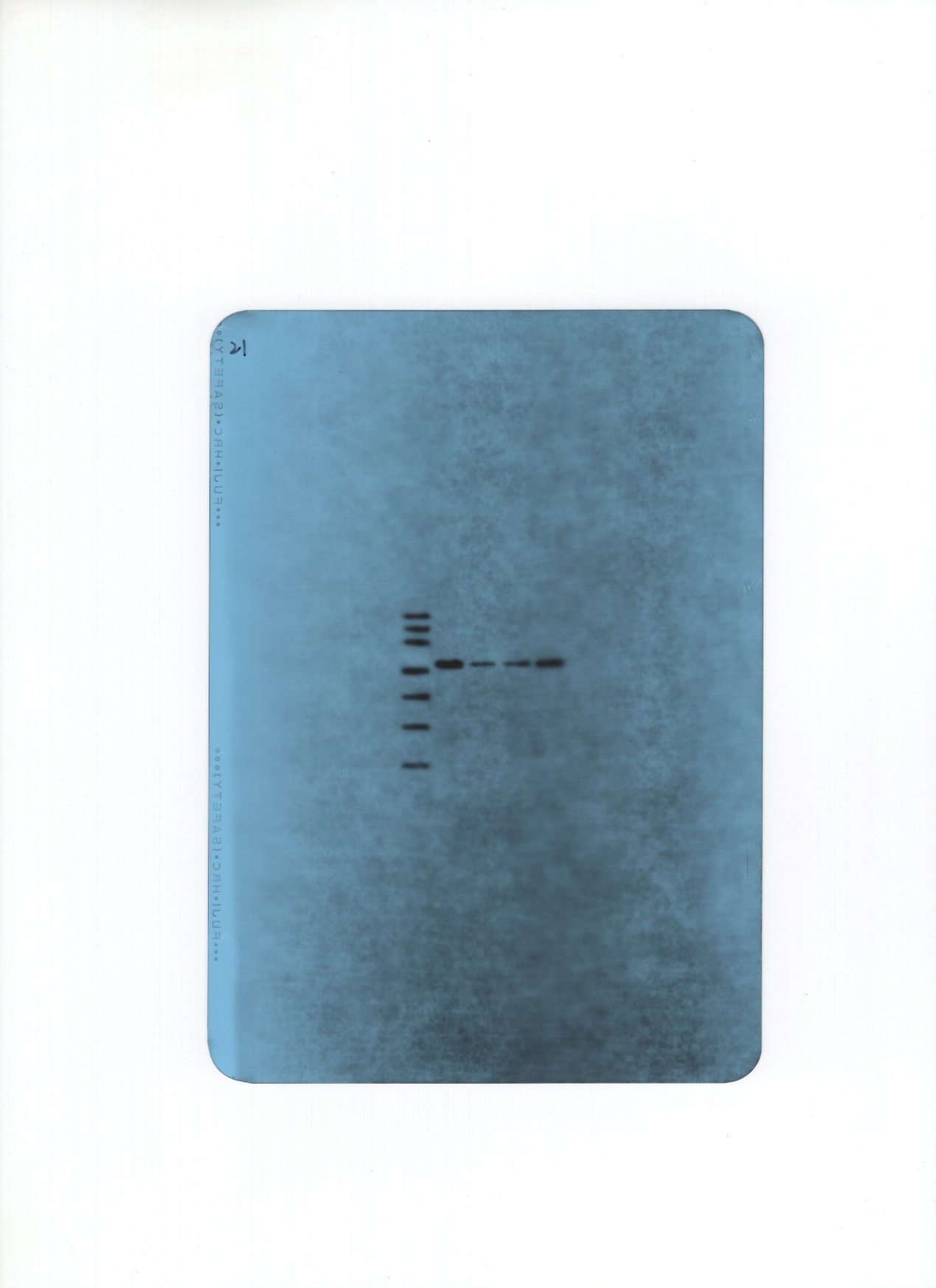


GAPDH


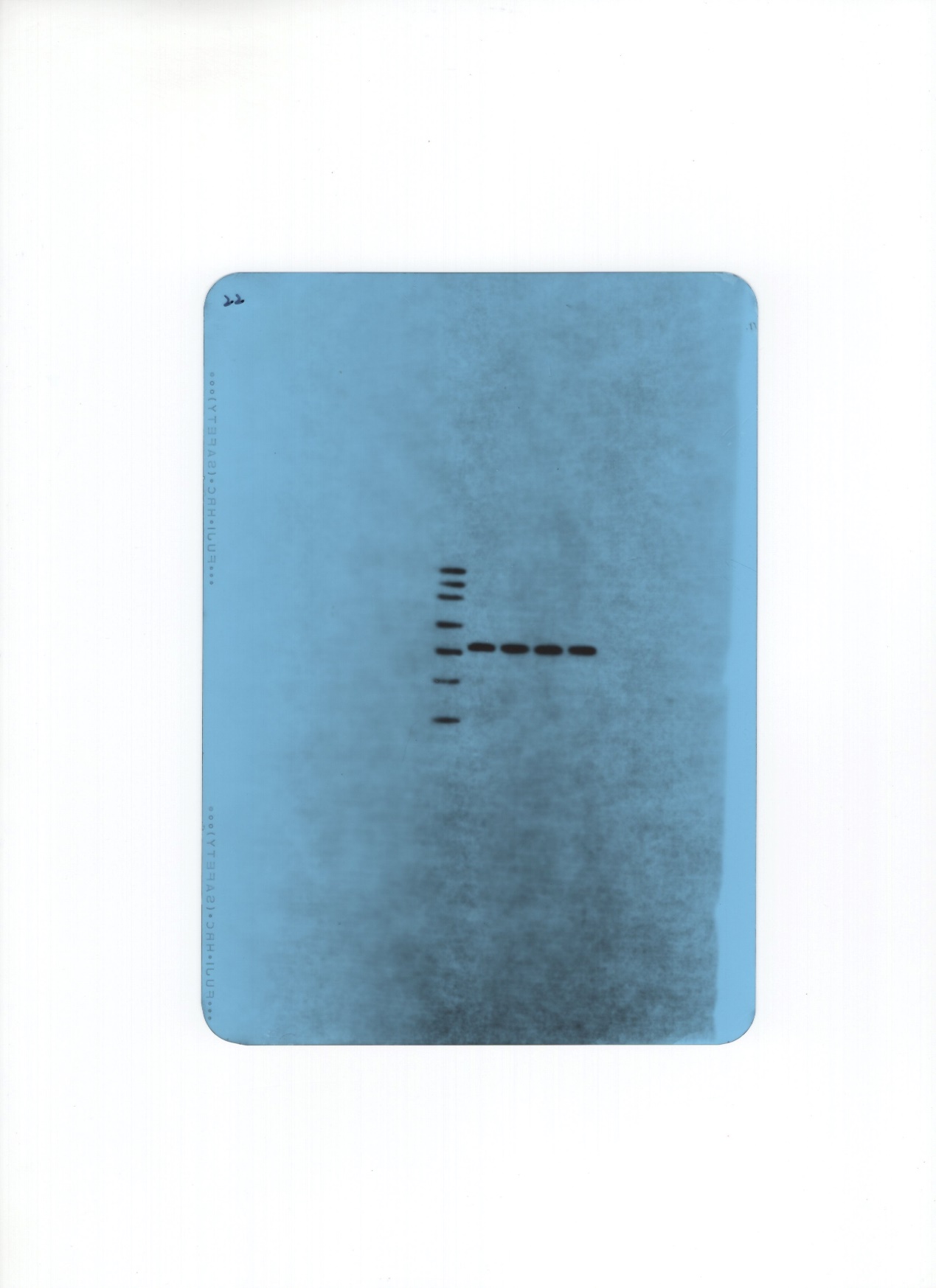


Figure 5E

BAX


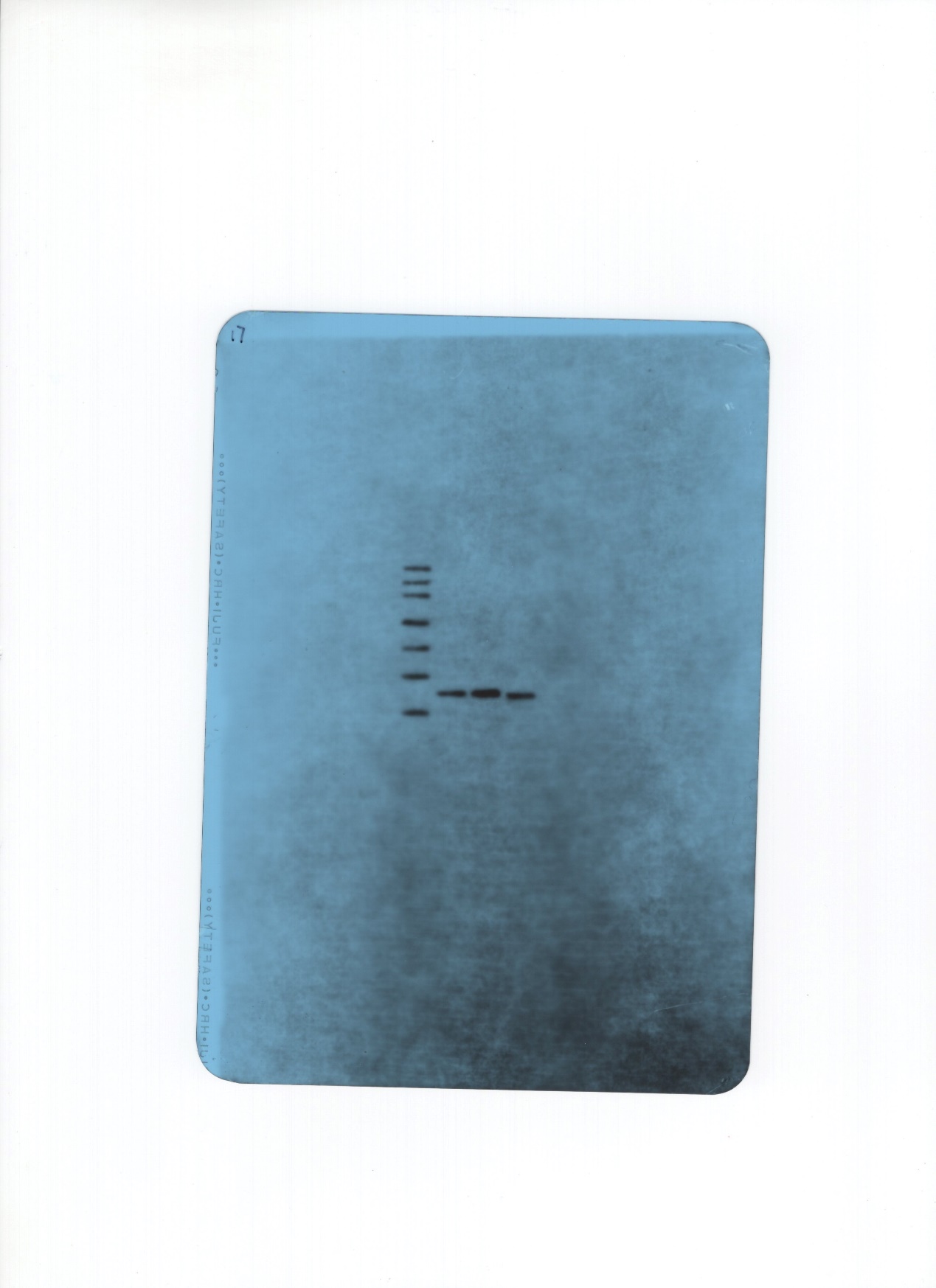


Bcl-2


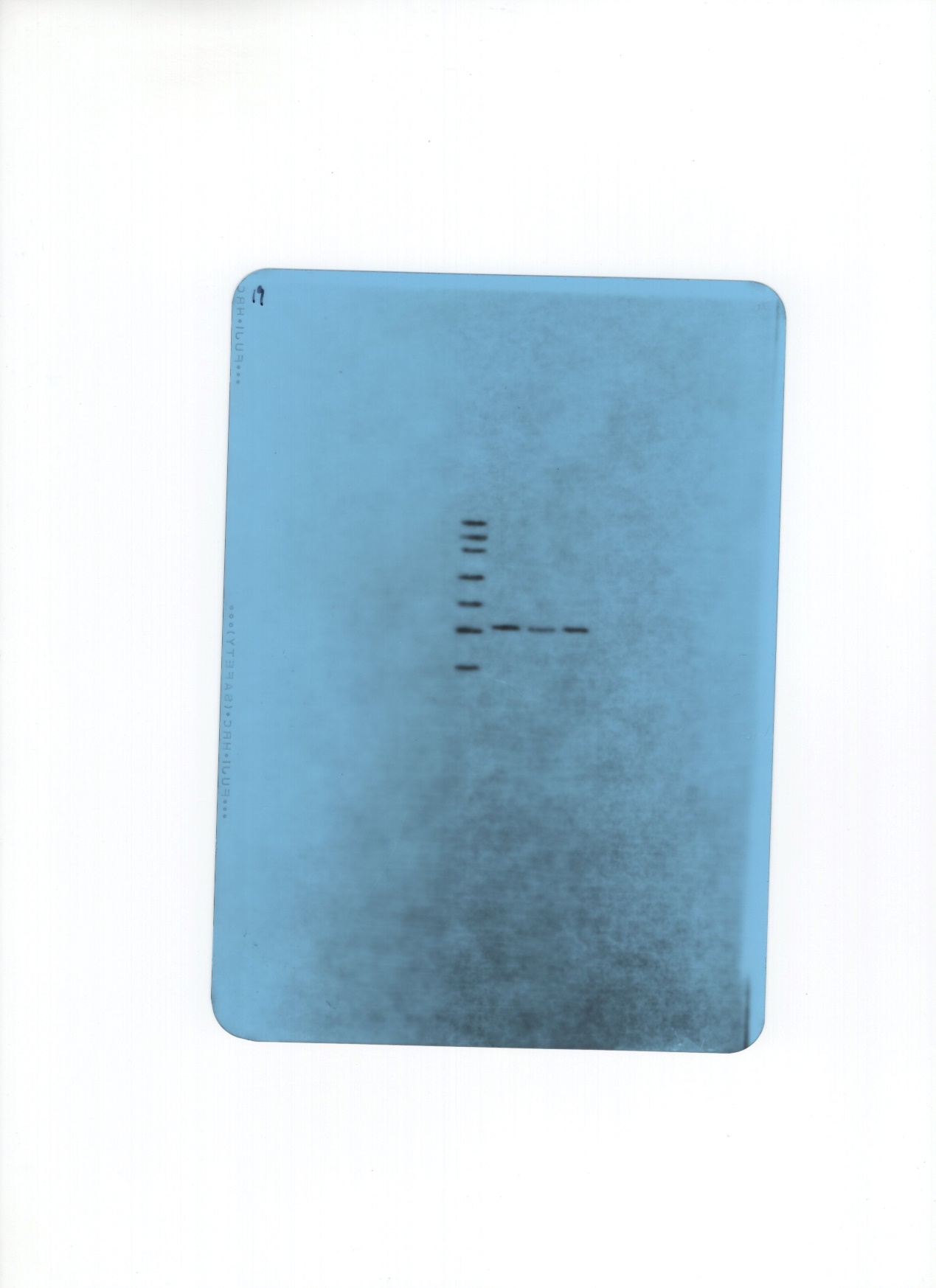


cleaved-caspase 3


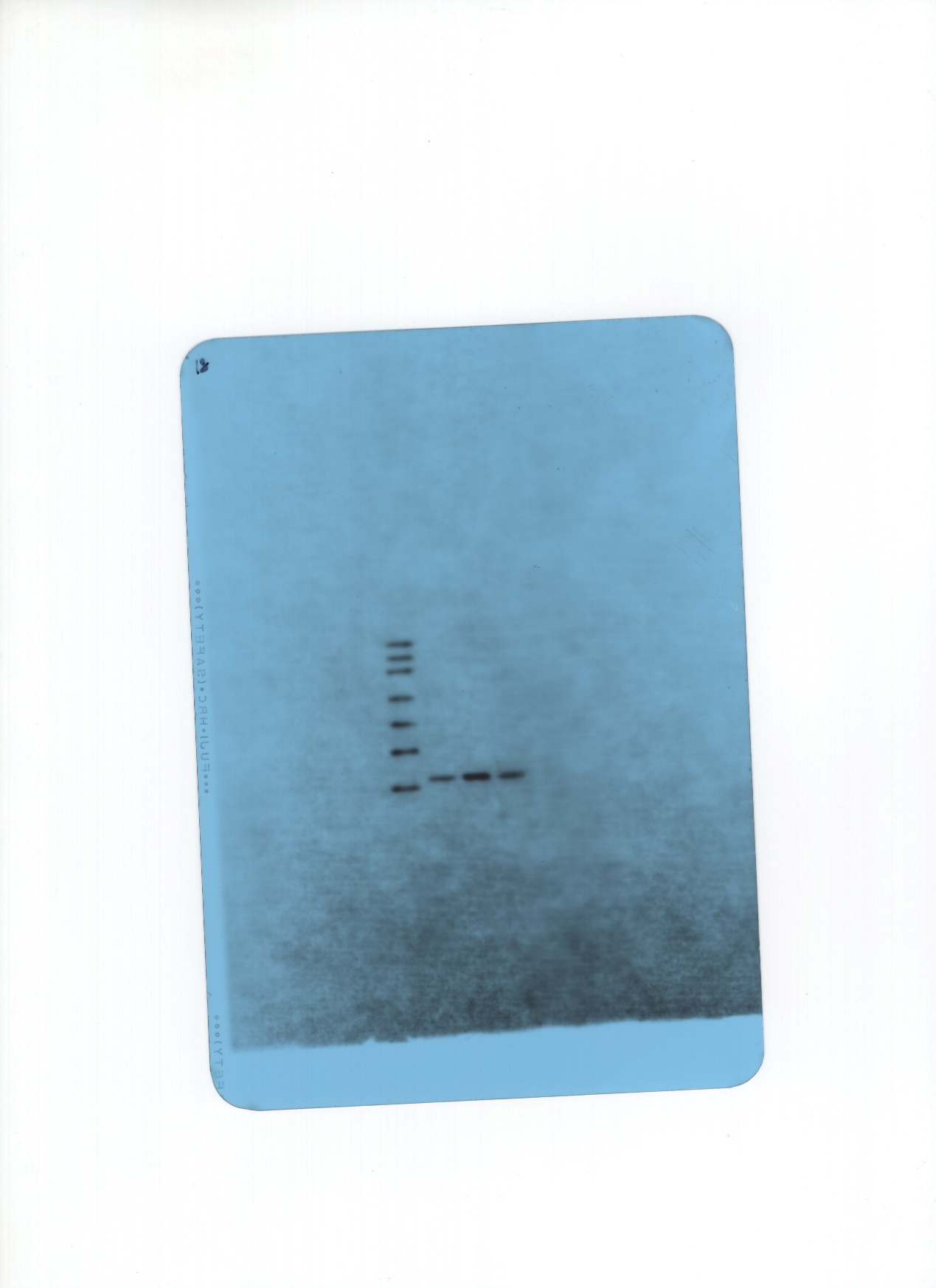


GAPDH


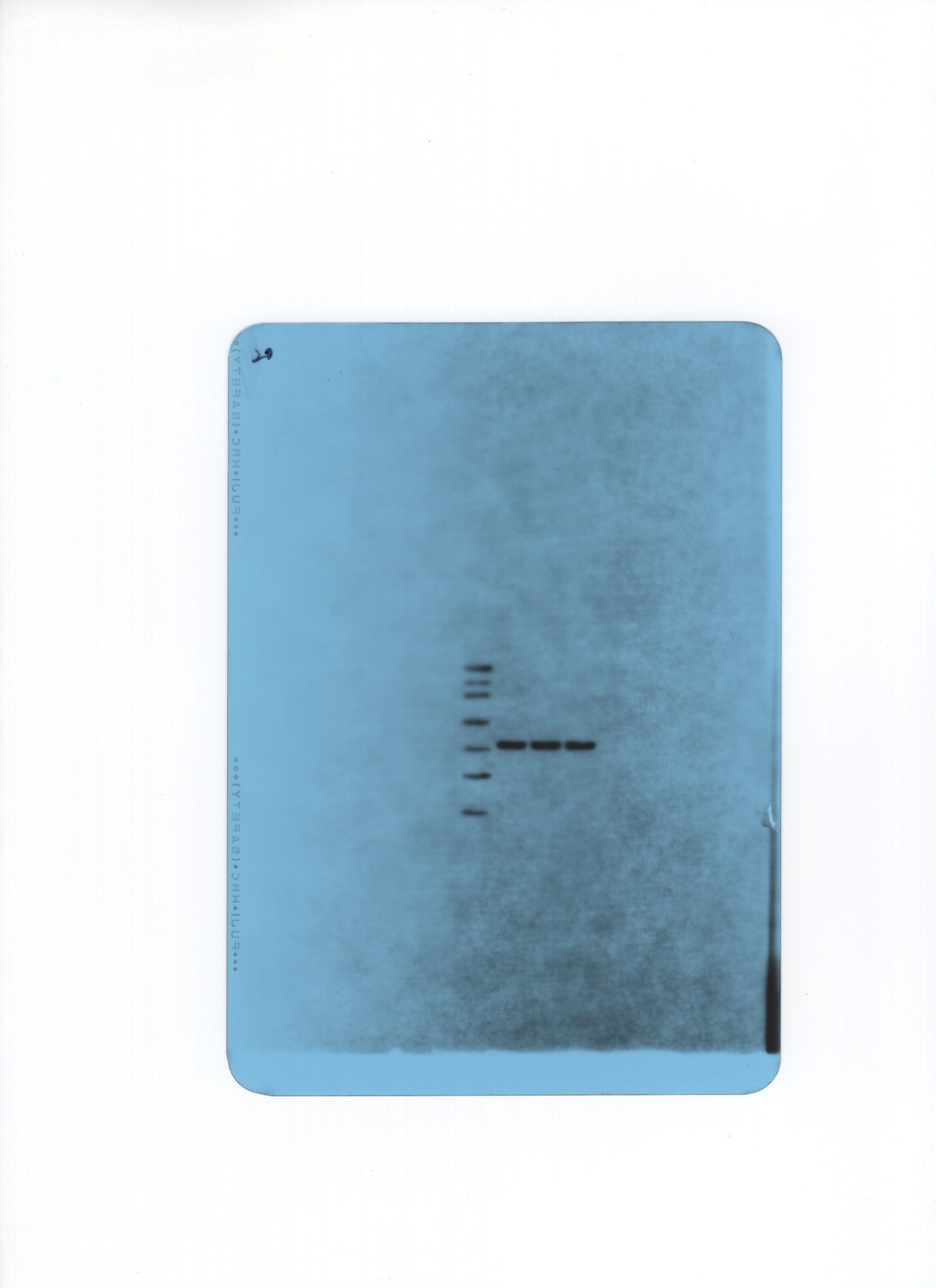


Figure 5F

BAX


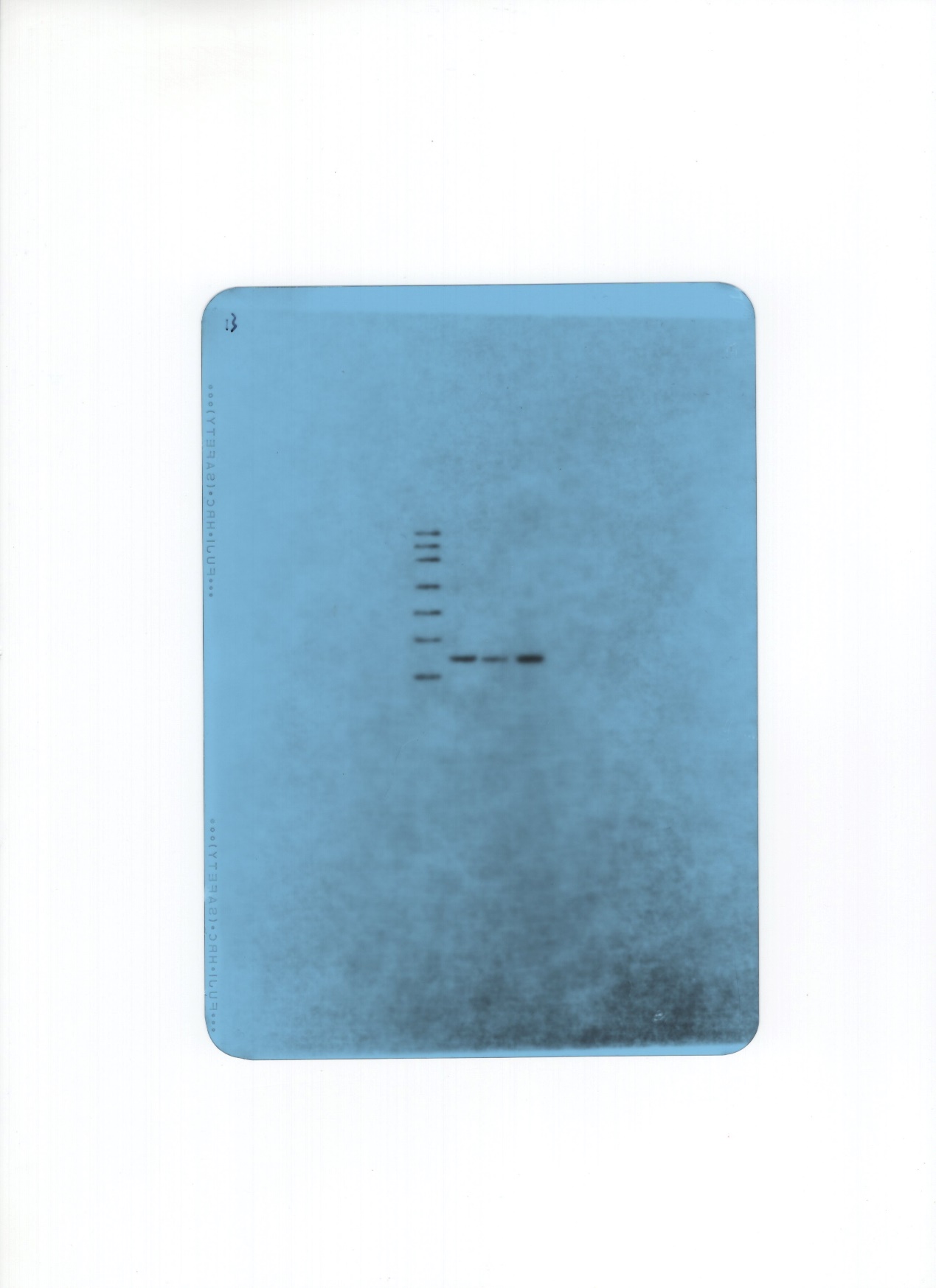


Bcl-2


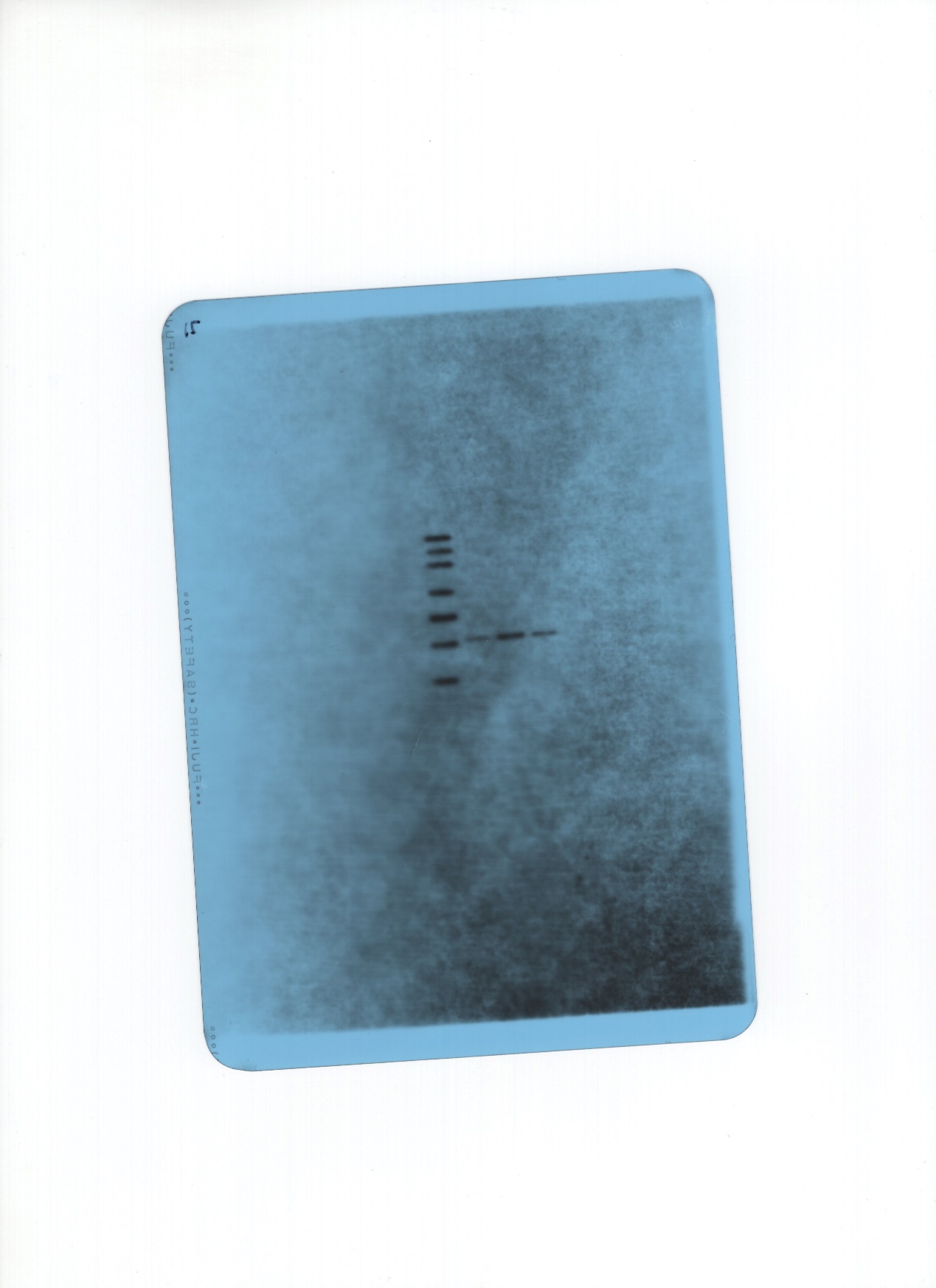


cleaved-caspase 3


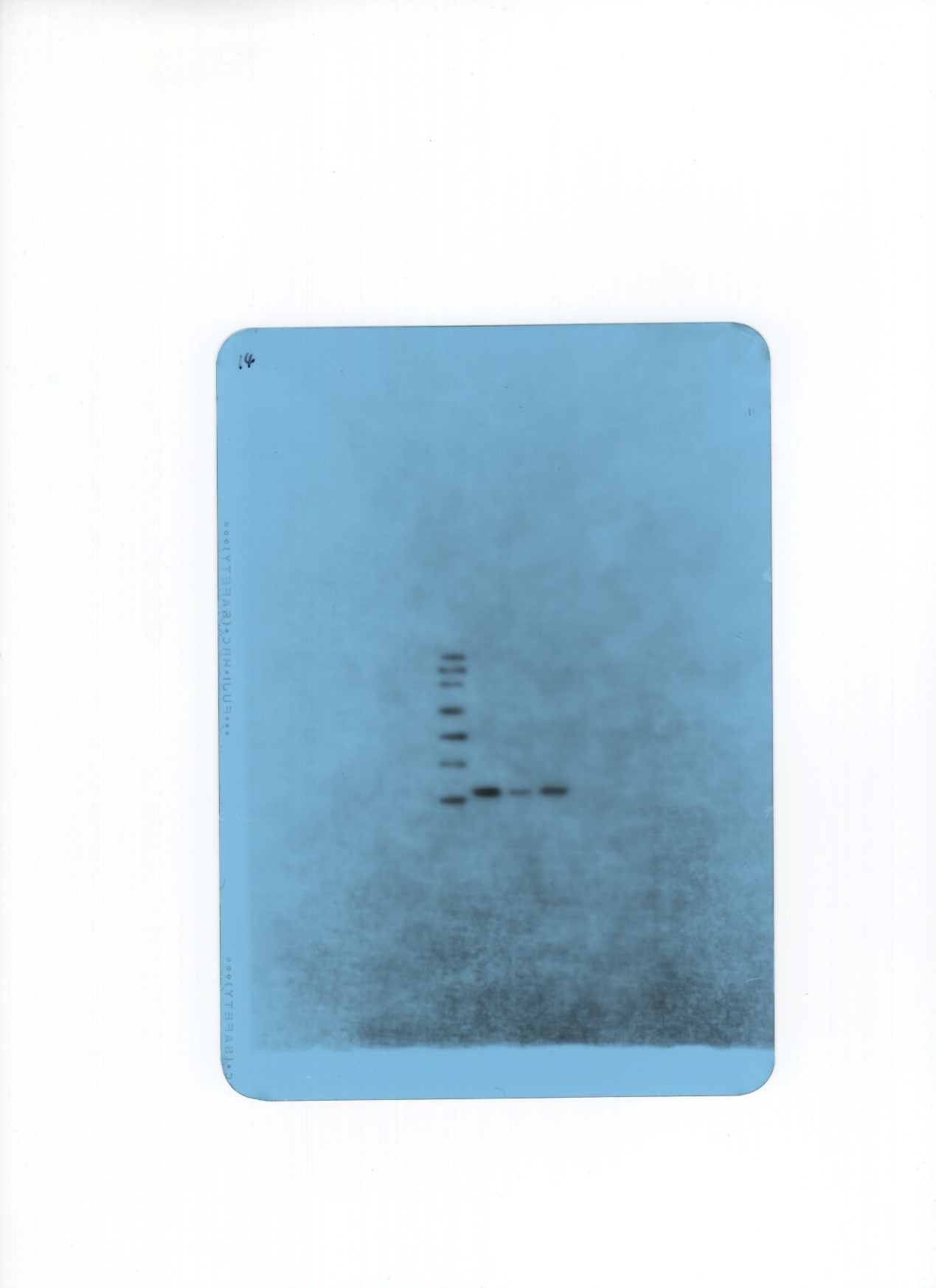


GAPDH


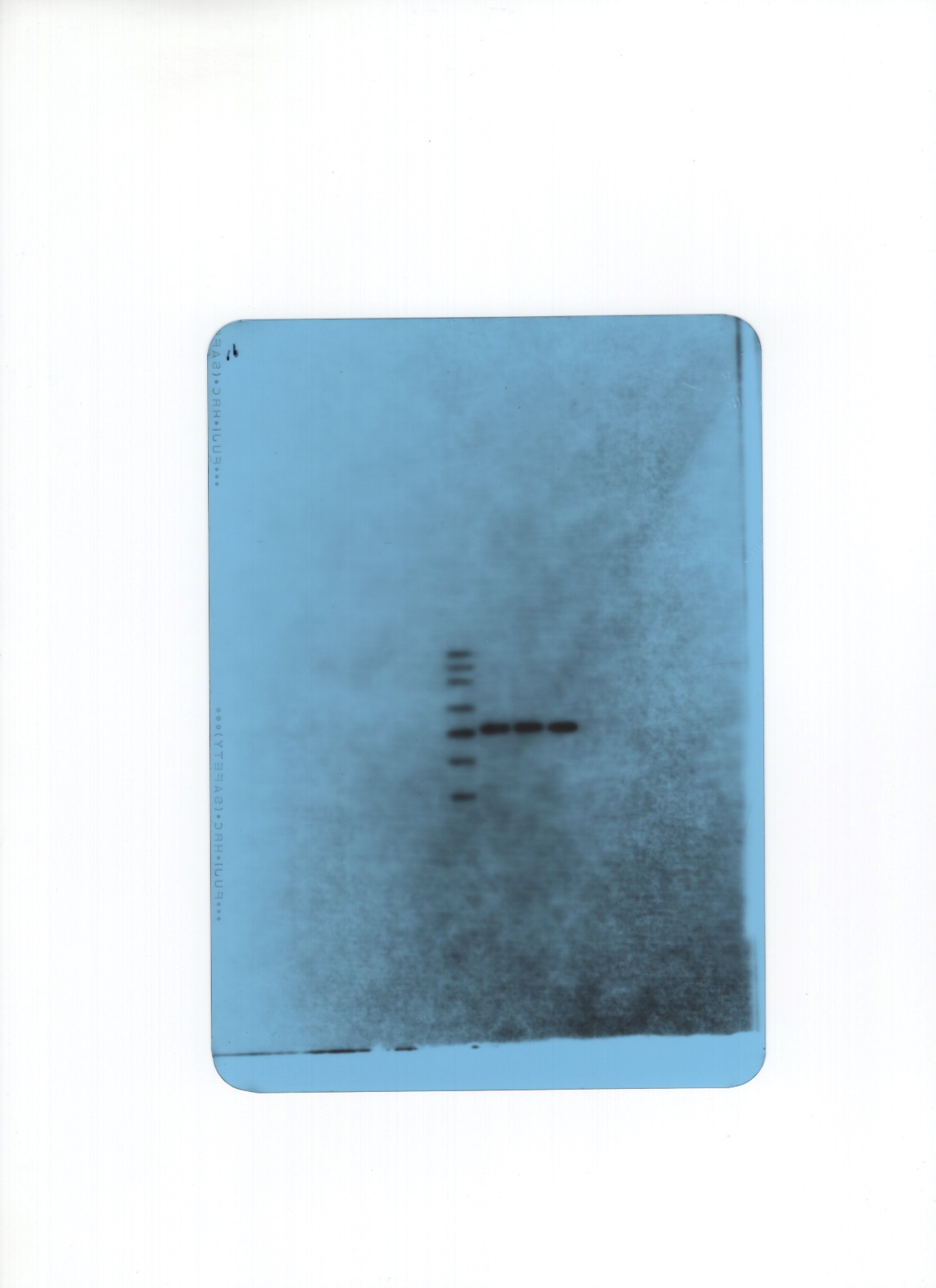

Supplement: Supplementary file 1 — Additional file 1. [file 12885_2022_10188_MOESM1_ESM.docx]
